# Differential Transcriptional Responses in Two Old World *Bemisia tabaci* Cryptic Species Post Acquisition of Old and New World Begomoviruses

**DOI:** 10.3390/cells11132060

**Published:** 2022-06-29

**Authors:** Habibu Mugerwa, Saurabh Gautam, Michael A. Catto, Bhabesh Dutta, Judith K. Brown, Scott Adkins, Rajagopalbabu Srinivasan

**Affiliations:** 1 Department of Entomology, University of Georgia, 1109 Experiment Street, Griffin, GA 30223, USA; habibu.mugerwa@uga.edu (H.M.); sg37721@uga.edu (S.G.); mac65630@uga.edu (M.A.C.); 2 Department of Plant Pathology, University of Georgia, 3250 Rainwater Road, Tifton, GA 31793, USA; bhabesh@uga.edu; 3 School of Plant Sciences, University of Arizona, Tuscon, AZ 85721, USA; jbrown@ag.arizona.edu; 4 USDA-ARS, U.S. Horticultural Research Laboratory, Fort Pierce, FL 34945, USA; scott.adkins@usda.gov

**Keywords:** cucurbit leaf crumple virus, sida golden mosaic virus, tomato yellow leaf curl virus, Middle East-Asia Minor 1 whitefly, Mediterranean whitefly, transcriptomes, virus–vector interaction

## Abstract

Begomoviruses are transmitted by several cryptic species of the sweetpotato whitefly, *Bemisia tabaci* (Gennadius), in a persistent and circulative manner. Upon virus acquisition and circulative translocation within the whitefly, a multitude of molecular interactions occur. This study investigated the differentially expressed transcript profiles associated with the acquisition of the Old World monopartite begomovirus, tomato yellow leaf curl virus (TYLCV), and two New World bipartite begomoviruses, sida golden mosaic virus (SiGMV) and cucurbit leaf crumple virus (CuLCrV), in two invasive *B. tabaci* cryptic species, Middle East-Asia Minor 1 (MEAM1) and Mediterranean (MED). A total of 881 and 559 genes were differentially expressed in viruliferous MEAM1 and MED whiteflies, respectively, compared with their non-viruliferous counterparts, of which 146 genes were common between the two cryptic species. For both cryptic species, the number of differentially expressed genes (DEGs) associated with TYLCV and SiGMV acquisition were higher compared with DEGs associated with CuLCrV acquisition. Pathway analysis indicated that the acquisition of begomoviruses induced differential changes in pathways associated with metabolism and organismal systems. Contrasting expression patterns of major genes associated with virus infection and immune systems were observed. These genes were generally overexpressed and underexpressed in *B. tabaci* MEAM1 and MED adults, respectively. Further, no specific expression pattern was observed among genes associated with fitness (egg production, spermatogenesis, and aging) in viruliferous whiteflies. The weighted gene correlation network analysis of viruliferous *B. tabaci* MEAM1 and MED adults identified different hub genes potentially implicated in the vector competence and circulative tropism of viruses. Taken together, the results indicate that both vector cryptic species and the acquired virus species could differentially affect gene expression.

## 1. Introduction

*Bemisia tabaci* (Gennadius) (Hemiptera: Aleyrodidae), commonly known as the sweetpotato whitefly, is widely distributed in the subtropics/tropics, where it can be an agricultural pest [1,2,3]. Feeding by this pest induces physiological symptoms and results in the transmission of a devastating group of plant viruses belonging to the genus *Begomovirus* [4,5,6,7,8]. *Bemisia tabaci* comprises several cryptic species with distinct phylogeographical distributions [9,10,11,12,13]. These cryptic species and their haplotypes are morphologically indistinguishable from one another, and some have been found to possess distinct biological traits such as fecundity, endosymbiont complement, host range, the propensity to develop insecticide resistance, ecological adaptation, and, importantly, transmission specificity involving begomoviruses [1,14,15,16,17,18]. 

Begomoviruses are small non-enveloped viruses with single-stranded circular DNA genomes of about 2800 (monopartite) to 5200 (bipartite) nt each, and they possess a unique morphology consisting of twinned or paired icosahedral particles [5]. Monopartite viruses such as TYLCV consist of a single DNA component, and bipartite viruses such as SiGMV and CuLCrV include both DNA-A and DNA-B components [5,19,20,21]. For the successful transmission of begomoviruses, whiteflies must ingest viral particles with their stylets while feeding. Virions pass through the food canal and reach the esophagus and midgut, where they traverse into the hemolymph. Virions are then endocytosed into the primary salivary glands and are egested with the saliva into the plant phloem. This circulative translocation of begomoviruses in *B. tabaci* is dependent upon the viral coat protein interactions with probable receptors and other whitefly proteins [22]. Few receptors and begomovirus-interacting proteins such as the GroEL chaperone protein, heat shock proteins, midgut proteins, peptidyl-prolyl isomerase protein genes, and the peptidoglycan recognition protein gene have been identified [23,24,25,26,27]. However, there may be many others involved, and unravelling them is critical to understanding virus transmission by whiteflies.

Among the members of the *B. tabaci* species complex, the Middle East-Asia Minor 1 (MEAM1), also known as the North Africa-Middle East mitotype or B biotype, and the Mediterranean (MED), also known as the North Africa-Mediterranean mitotype or Q biotype, are invasive and widely distributed [2,10,11,12]. *Bemisia tabaci* MEAM1 was first reported in the United States in the mid-1980s and has since become the predominant cryptic species on field crops after displacing the native New World 1 species, also known as the American Tropics (AMTROP) cryptic species or A biotype, whereas *B. tabaci* MED was first reported in 2004 and is primarily confined to greenhouse-grown ornamentals [1,15,28]. Recently, *B*. *tabaci* MED was reported on open-field crops in Florida and Georgia [29,30]. In the southeastern United States, *B. tabaci* MEAM1 occurs on field-grown vegetable crops such as tomato, eggplant, snap bean, and cucurbits and on cotton [30,31]. In these cropping systems, *B. tabaci* MEAM1 is known to transmit tomato yellow leaf curl virus (TYLCV), sida golden mosaic virus (SiGMV), and cucurbit leaf crumple virus (CuLCrV), which cause diseases [32,33]. In laboratory studies, *B. tabaci* MED tissues accumulated reduced amounts of the two New World begomoviruses compared to *B. tabaci* MEAM1 and did not transmit SiGMV and CuLCrV, albeit it was an efficient vector of TYLCV [34,35,36]. Molecular and cellular factors underlying the differential transmission of these and several other begomoviruses by the *B. tabaci* MEAM1 and MED have not been elucidated, and studies identifying their primary transmission determinants are critical.

Plant viruses can alter the phenotype and physiological traits of their hosts, which in turn modulates vector preference and fitness, at times favoring their spread [37,38]. Begomovirus infections were reported to influence vector preference and fitness in different ways depending on the host plant [39,40,41,42]. However, the mechanisms involved in such begomoviruses-induced macro-effects on their whitefly vectors are not well understood, and high throughput sequencing platforms have been utilized to understand virus-associated macro-effects on whiteflies [43].

Using various omics platforms, including transcriptomics, the virus-associated micro-effects of begomoviruses and criniviruses have been examined for *B. tabaci* (MEAM1 and MED) [44,45,46,47,48,49,50,51,52,53,54]. However, what remains unclear is whether micro-effects are due to the direct effect of the virus on the vector or to the indirect effects resulting from the modulation of the physiological changes in the infected host plant following virus infection. To minimize the host-modulated indirect effects of plant viruses on their vectors through feeding on the plant sap of virus-infected hosts, it is crucial to transfer viruliferous whiteflies from a virus-infected host plant to a virus non-host plant for gut clearing prior to RNA extraction [44].

In this study, differential gene expression in *B. tabaci* MEAM1 and MED was assessed by transcriptome analysis post acquisition of three begomoviruses, the Old World monopartite TYLCV, and the New World bipartite SiGMV and CuLCrV. Libraries were prepared for viruliferous and non-viruliferous whiteflies given a 72 h acquisition access period (AAP) on the respective begomovirus-infected or non-infected host plants, followed by a 72 h feeding access on cotton, a non-host of the three begomoviruses. The objectives of this study were to (i) assess the differences in gene expression between *B. tabaci* MEAM1 and MED upon the acquisition of Old and New World begomoviruses, and (ii) locate putative hub genes and co-expressed genes (modules) responsive to the acquisition of Old and New World begomoviruses using weighted gene correlation network analysis (WGCNA).

## 2. Materials and Methods

### 2.1. Generation of Plants and Insect Rearing

Cotton, *Gossypium hirsutum* L. cv. PHY 339 WRF (UGA extension services, Tifton, GA, USA), tomato, *Solanum lycopersicum* cv. Florida 47 (Seminis Vegetable Seeds, St. Louis, MO, USA), prickly sida, *Sida spinosa* (grown from field-collected seeds from UGA, Tifton, GA, USA), and squash, *Cucurbita pepo* cv. Goldstar (Syngenta^®^, Wilmington, DE, USA) seeds were planted in 10 cm diameter × 8 cm tall pots (Hummert International, Earth City, MO, USA) at two seeds of each host per pot using the PRO-MIX BX general purpose medium (PRO-MIX, Quakertown, PA, USA). Fertigation was carried out at weekly intervals using water-soluble fertilizer (Miracle-Gro^®^, Scotts Miracle-Gro products, Inc., Marysville, OH, USA). Pots were placed in whitefly-proof cages (Megaview Science Co., Taichung, Taiwan) [47.5(l) × 47.5(w) × 93(h) cm^3^] in the greenhouse and maintained at 25 °C, 60% RH, and a 16 h L:8 h D photoperiod. One week post germination, the seedlings were thinned to one per pot. Cotton plants were used to rear whiteflies, as they are not a host of TYLCV, SiGMV, or CuLCrV.

The *B*. *tabaci* MEAM1 colony (Genbank accession number: MN970031) was established from individuals collected in 2009 from cotton plants in Tifton, Georgia. This colony was reared on cotton plants in 10 cm diameter × 8 cm tall pots in whitefly-proof cages in the greenhouse under the conditions described above. The *B*. *tabaci* MED colony (GenBank accession number: MZ469725) was established on cotton plants in 2017 from individuals collected from poinsettia (*Euphorbia pulcherrima* Wild. Ex Klotzsch) plants from a nursery located in North Georgia, USA and maintained by Prof. Ronald D. Oetting, College of Agriculture and Environmental Sciences, University of Georgia, Griffin, GA. The *B*. *tabaci* MED colony was reared under the same conditions as those reported for the *B. tabaci* MEAM1 colony. The identities of both colonies were examined by PCR amplification and by partially sequencing the mitochondrial cytochrome oxidase I (COI) gene in the 3’ barcode region [55,56]. The *B*. *tabaci* MEAM1 and MED colonies were maintained in separate greenhouses to avoid population admixtures, and their identities were periodically screened (every alternate month) to ensure no contamination had occurred.

The virus isolates used in this study are described extensively in Gautam et al. [36]. Briefly, the TYLCV isolate was collected in 2009 from a commercial tomato farm located in Montezuma in Macon County in GA, USA. TYLCV has since been maintained in TYLCV-susceptible tomato cultivar (Florida 47) through *B. tabaci* MEAM1-mediated inoculation. The SiGMV isolate was collected in 2018 from prickly sida plants on field edges at the UGA Horticulture Hill Farm in Tifton (Tift County, GA, USA). SiGMV has since been maintained in prickly sida plants through *B. tabaci* MEAM1-mediated inoculation. The CuLCrV isolate used in this study was collected in 2016 from infected yellow summer squash plants from the UGA Horticulture Hill Farm in Tifton (Tift County, GA, USA). CuLCrV has since been maintained in yellow summer squash plants through *B. tabaci* MEAM1-mediated inoculation. Virus-infected plants were obtained by providing *B. tabaci* MEAM1 a 72 h acquisition access period (AAP) on TYLCV/SiGMV/CuLCrV-infected plants. Subsequently, viruliferous *B. tabaci* MEAM1 (~100/plant) were attached to the non-infected ~10 cm tall seedlings (tomato, squash, or prickly sida) using clip cages. Inoculated individual plants were placed in separate insect-proof cages under the conditions described above. A week post inoculation, all of the leaves were removed. The infection status of plants in insect-free newly-emerged leaves was evaluated at ~3 weeks post inoculation by endpoint PCR using species-specific primers [36,39].

### 2.2. Feeding Assay, DNA & RNA Isolation, and RNA Sequencing 

Approximately 1000 *B. tabaci* MEAM1 or MED newly emerged (1–3 days old) whiteflies were collected from the above established colonies and then introduced to either non-infected or virus-infected tomato, prickly sida, and squash plants for an acquisition access period (AAP) of 72 h. After 72 h on either non-infected or virus-infected tomato, prickly sida, or squash plants, the whiteflies (MEAM1 and MED) were transferred to cotton plants for another 72 h to minimize the host effect from either non-infected or infected plants. After 72 h on cotton, ~100 live whiteflies in three to five biological replicates (per treatment) were collected and immediately stored at −20 °C for 20 min and thereafter used for RNA extraction on the same day. Another set of 20 individual whiteflies (MEAM1 or MED) per treatment were collected and used to confirm the presence/absence of the virus. The results indicated that 90–100%, 80–90%, or 70–90% of the *B. tabaci* MEAM1 and 95–100%, 65–85%, or 60–80% of the MED whiteflies were positive for TYLCV, SiGMV, or CuLCrV, respectively. As expected, individual *B*. *tabaci* MEAM1 or MED that were given a 72 h feeding access on non-infected tomato, prickly sida, or squash plants (and for 72 h on cotton plants) tested negative for TYLCV, SiGMV, and CuLCrV, respectively.

The total whitefly DNA was extracted from individuals using an InstaGene Matrix containing 6% Chelex resin (Bio-Rad, Hercules, CA, USA), as previously described by Gautam et al. [30]. The extracted DNA was stored at −20 °C until it was used. PCR reactions containing 2X GoTaq Green Master Mix (Promega, Madison, WI, USA) and primers specific to TYLCV, SiGMV, or CuLCrV (Table S1) were performed using a T-100 thermocycler (Bio-Rad, Hercules, CA, USA). The total RNA was extracted using the Ambion TRIzol Reagent (Thermo Fisher, Waltham, MA, USA), according to the manufacturer’s instructions, and purified using the PureLink^TM^ RNA Mini Kit (Thermo Fisher, Waltham, MA, USA), according to the manufacturer’s instructions. Approximately 50 μL of RNA was shipped to Novogene Corporation Inc. (Sacramento, CA, USA), where RNA sample quality control (QC), mRNA library preparation, and RNA sequencing were carried out.

### 2.3. RNA Sequencing, Transcriptome Assembly, and Analysis

RNA sample quality control (QC) was carried out using a Nanodrop for preliminary quantitation. Agarose gel electrophoresis was used to test RNA degradation and potential contamination, while the Agilent 2100 was used to assess RNA integrity (RIN) and quantitation. The samples that passed QC (RIN value > 6.8 and concentration of >20 ng/uL) were used for library construction. In brief, the library construction involved enriching mRNA using oligo(dT) beads and the removal of rRNA using the Ribo-Zero kit. Subsequently, the mRNA was fragmented and followed by first- and second-strand cDNA synthesis. Finally, cDNA libraries were generated through adaptor ligation and PCR enrichment. The library QC consisted of three steps: testing library concentration using Qubit 2.0, testing the insert size using the Agilent 2100, and quantifying the library concentration using qPCR. The libraries that passed QC were sequenced on a NovaSeq 6000 Sequencing System (Illumina, San Diego, CA, USA) using the NovaSeq paired end 150 sequencing setting.

Trim Galore v0.6.5 (https://www.bioinformatics.babraham.ac.uk/projects/trim_galore/ accessed on 1 December 2021) was used to remove adaptors and low-quality sequences with fewer than 20 nt from the high-quality RNA-Seq reads. The quality filtering parameter was set to the default threshold of 20 on a phred+33 scale, where the average quality score per read was 36 across all samples. Ribosomal RNA, the assembled *B. tabaci* mitochondrion genome, and three bacterial endosymbiont genomes of *Candidatus Portiera aleyrodidarum*, *Hamiltonella*, and *Rickettsia* were aligned to the cleaned reads using BLAT v3.5 [51,57,58,59]. The aligned reads were removed using Seqtk v1.3 (https://github.com/lh3/seqtk accessed on 3 December 2021). The remaining high-quality cleaned paired-end reads were aligned to the reference *B. tabaci* genome using STAR v2.7.2 [51,60]. Differentially expressed genes between viruliferous (TYLCV/SiGMV/CuLCrV) and non-viruliferous whiteflies were identified using edgeR v3.28.1 [61]. Multiplicity correction was performed by applying the Benjamini–Hochberg method on the *p*-values to control the false discovery rate (FDR) [62]. For each comparison, genes with an adjusted *p*-value < 0.05 and a fold change (ratio of viruliferous/non-viruliferous whiteflies) ≥ 1.4 were considered as significantly differentially expressed genes. Volcano plots were generated in R software v4.1.0, and differentially expressed genes were assigned to gene ontology (GO) classes and Kyoto Encyclopedia of Genes and Genomes (KEGG) pathways using Blast2GO (BioBam Bioinformatics, Valencia, Spain) and BlastKOALA (http://www.kegg.jp/blastkoala/ accessed on 15 December 2021), respectively [63,64,65]. Significantly expressed GO classes were summarized by removing redundant terms and visualized using REVIGO (http://revigo.irb.hr/ accessed on 10 June 2022) [66]. The annotations of *B. tabaci* genes were obtained from the published MEAM1 genome for a parallel comparison between *B. tabaci* MEAM1 and MED transcriptomes [51]. Venn diagrams were created using a webtool from Bioinformatics and Evolutionary Genomics, Ghent University (http://bioinformatics.psb.ugent.be/webtools/Venn/ 21 December 2021) and normalized on percentage basis.

The weighted gene co-expression network (WGCNA) software v1.70-3 was run using the R software v4.1.0 [65,67]. Prior to the construction of the co-expression networks, filtering was performed to select the top 95% quantile of DEGs from the 25 and 27 samples of *B. tabaci* MEAM1 and MED, respectively. The soft-thresholding power modules (1 to 40) was tested by the gradient independent method, and the scale-independent condition of the signed R2 was set to 0.90. Gene modules were constructed with a power of 28, with a minimum module size set to 20. The interaction relationships across the co-expression modules were used to construct a topological overlap matrix (TOM) by using correlation expression values. The TOM was represented with a dendrogram by setting the parameters mergeCutHeight = 0.15 and detectCutHeight = 0.995. The module color was randomly assigned, and the first principal component from each module was used to calculate the module eigengene. A module–trait relationship heatmap was generated using the labeledHeatmap package available in WGCNA software to show the topological overlap of the co-expression modules based on the module eigengenes. The labeledHeatmap parameters included matrix = moduleTraitCor and main = paste (“Module-trait relationships”). The network analysis of the top 30 genes from the *B*. *tabaci* MEAM1 and MED turquoise modules was visualized with Cytoscape v3.9.0 [68].

### 2.4. Validation of RNA Sequencing Data

To validate the differential expression analysis, 10 genes were randomly selected for each virus treatment (*n* = 56), and their expression levels were compared between viruliferous and non-viruliferous whiteflies (*B. tabaci* MEAM1 or MED) for the three viruses (TYLCV, SiGMV, and CuLCrV). For each sample, an aliquot (10 μL) of the RNA was used for validation by reverse transcription quantitative PCR (RT- qPCR), and the remainder were used for RNA Sequencing. The primer sequences used for the selected genes are listed in Table S1. The total RNA for each sample (500 ng) was reverse transcribed into cDNA using the GoScript^TM^ Reverse Transcription System (Promega, Madison, WI, USA), according to the manufacturer’s instructions. Quantitative PCR reactions were assembled in 12.5 μL duplicate reactions for three biological replicates and carried out using 2X GoTaq qPCR Master Mix (Promega, Madison, WI, USA) in a Mastercycler^®^ ep realplex thermal cycler (Eppendorf, Hauppauge, NY, USA). An initial denaturation step (2 min at 94 °C) was followed by 40 cycles of denaturation (20 s at 94 °C), primer annealing (15 s at 58–60 °C, depending on the primer set), and extension (15 s at 68 °C). Finally, the melting curve analysis was conducted to evaluate the specificity of the fluorescence signal. Relative expression levels were calculated using the 2^−ΔΔCt^ method, and the expression level of each gene was normalized to the expression level of β-actin, a whitefly reference gene [69,70].

## 3. Results

### 3.1. Summary of RNA Sequencing

Three to five biological replicates were included per virus treatment, resulting in a total of 25 and 27 libraries constructed for *B. tabaci* MEAM1 and MED adults, respectively. Raw read pairs for the generated libraries ranged from ~19 to 33 million. After trimming and removing the reads that aligned with the ribosomal RNA, the mitochondrion genome, and the three bacterial endosymbiont genomes (*Candidatus Portiera aleyrodidarum*, *Hamiltonella*, and *Rickettsia*), ~17 to 31 million read pairs were retained in the libraries (Table 1). *Bemisia tabaci* MEAM1 and MED cleaned read pairs for the different libraries mapped onto the *B. tabaci* MEAM1 genome by ~90 to 95% and 88 to 91%, respectively (Table 1). The Pearson’s correlation coefficients for all of the biological replicates ranged from 0.9 to 1 (Table S2), suggesting that the data were highly reproducible.

### 3.2. Overview of Differentially Expressed Genes in B. tabaci MEAM1 and MED Adults

**Figure 1:**
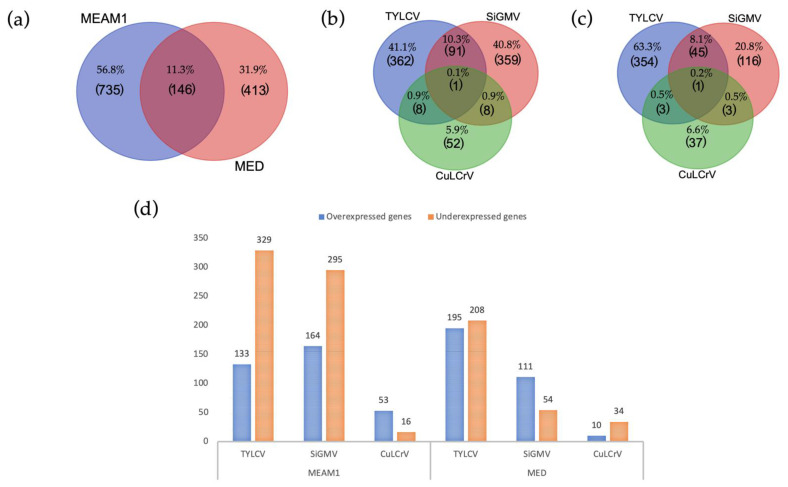
Differentially expressed genes (DEGs) in *Bemisia tabaci* Middle East-Asia Minor 1 (MEAM1) and Mediterranean (MED) adults provided with an acquisition access period of 72 h on TYLCV-infected versus non-infected tomato plants, SiGMV-infected versus non-infected prickly sida plants, or CuLCrV-infected versus non-infected squash plants: (**a**) Normalized Venn diagram showing unique and common DEGs in *B*. *tabaci* MEAM1 and MED adults after the acquisition of TYLCV, SiGMV, or CuLCrV; (**b**) Normalized Venn diagram showing unique and common DEGs in *B*. *tabaci* MEAM1 adults after the acquisition of TYLCV, SiGMV, or CuLCrV; (**c**) Normalized Venn diagram showing unique and common DEGs in *B*. *tabaci* MED adults after the acquisition of TYLCV, SiGMV, or CuLCrV; (**d**) Number of DEGs in *B. tabaci* MEAM1 and MED detected between viruliferous (TYLCV, SiGMV, or CuLCrV ) and non-viruliferous insects.

**Figure 2:**
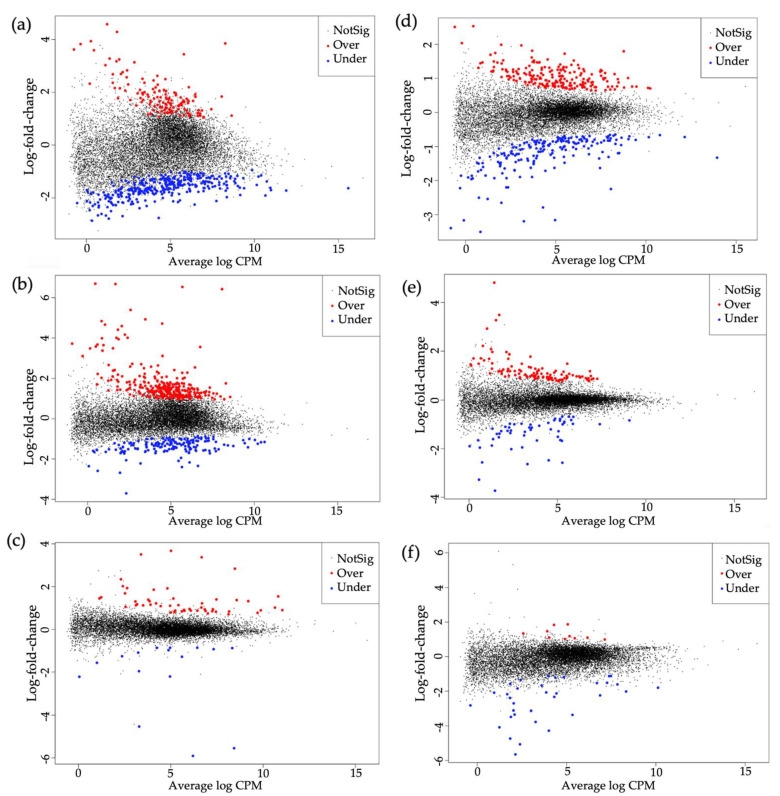
Mean difference plots of differentially expressed genes in *B*. *tabaci* Middle East-Asia Minor 1 (MEAM1) and Mediterranean (MED) adults provided with an acquisition access period of 72 h on virus-infected versus non-infected plants. Plots of log-intensity ratios (differences) versus log-intensity averages (means). CPM indicates counts per million reads. (**a**) *B*. *tabaci* MEAM1 adults with versus without TYLCV; (**b**) *B*. *tabaci* MEAM1 adults with versus without SiGMV; (**c**) *B*. *tabaci* MEAM1 adults with versus without CuLCrV; (**d**) *B*. *tabaci* MED adults with versus without TYLCV; (**e**) *B*. *tabaci* MED adults with versus without SiGMV; and (**f**) *B*. *tabaci* MED adults with versus without CuLCrV. Black, red, and blue dots are indicative of genes not significantly expressed, significantly overexpressed, or significantly underexpressed, respectively.

Of the 15,668 genes in the genome of *B. tabaci* MEAM1 (http://www.whiteflygenomics.org/cgi-bin/bta/index.cgi accessed on 15 December 2021), a total of 881 and 559 genes were differentially expressed in viruliferous *B. tabaci* MEAM1 and MED adults, respectively, when compared with non-viruliferous whiteflies (Figure 1a–c). In *B. tabaci* MEAM1 adults, 462 genes (133 overexpressed, 329 underexpressed), 459 genes (164 overexpressed, 295 underexpressed), and 69 genes (53 overexpressed, 16 underexpressed) were differentially expressed following the acquisition of TYLCV, SiGMV, or CuLCrV, respectively (Figure 1d and Figure 2a–c). In *B. tabaci* MED adults, 403 genes (195 overexpressed, 208 underexpressed), 165 genes (111 overexpressed, 54 underexpressed), and 44 genes (10 overexpressed, 34 underexpressed) were differentially expressed following the acquisition of TYLCV, SiGMV, or CuLCrV, respectively (Figure 1d and Figure 2d–f).

The RNA sequencing-based differential gene expression results were validated via RT-qPCR on 10 randomly selected differentially expressed genes per whitefly species-virus treatment (*n* = 56), using the β-actin gene as an internal standard (Table S3). The results showed that the expression trends obtained from RNA sequencing and RT-qPCR were highly consistent (Table S3).

### 3.3. Common DEGs among B. tabaci MEAM1 and MED Adults

**Figure 3:**
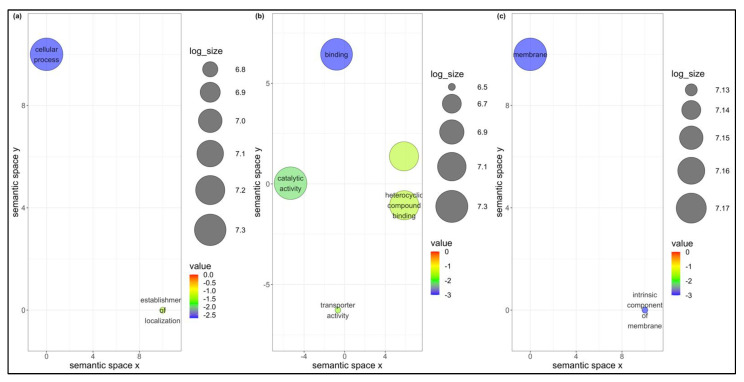
Scatterplots showing (**a**) biological process, (**b**) molecular function, and (**c**) cellular component gene ontology terms common between *Bemisia tabaci* Middle East-Asia Minor 1 and Mediterranean following the acquisition of viruses. Cluster representatives in a two-dimensional space were derived by applying multidimensional scaling to a matrix of the gene ontology terms’ semantic similarities. Bubble color indicates the *p*-value, and size indicates the frequency of the GO term in the underlying GOA database.

Of the 881 and 559 DEGs found in *B. tabaci* MEAM1 and MED adults, respectively, 146 were found to be differentially expressed in common following the acquisition of the three viruses (Figure 1a). Seventy genes—almost half of the common DEGs between *B. tabaci* MEAM1 and MED adults—were unknown, whereas the other 76 genes were annotated (http://www.whiteflygenomics.org/cgi-bin/bta/index.cgi accessed on 15 December 2021). All of the common DEGs (146) between *B. tabaci* MEAM1 and MED adults were analyzed using functional analysis tools (Blast, InterProScan, Blast2GO Mapping, and Blast2GO Annotation) available in the OmicsBox version 1.4.11. Only 72 genes (69 annotated and 3 unknown) were assigned functional groups under three classification systems: biological process (44 genes), molecular function (60 genes), and cellular component (33 genes). Fourteen Gene Ontology (GO) terms were assigned under the biological process category, of which the cellular process and the establishment of localization were significant (Figure 3a). Eight GO terms were assigned under the molecular function category, of which catalytic activity, binding, heterocyclic compound binding, and transport activity were significant (Figure 3b). In the cellular component category, two GO terms were significant, and they were membrane and intrinsic component of membrane (Figure 3c).

Only 28.8% (42 of 146) of the total number of common DEGs between *B*. *tabaci* MEAM1 and MED adults were annotated using the KEGG analysis [63] and mapped to 74 biochemical pathways (Table S4a,b). The most represented category pathways were (i) metabolism, (ii) organismal systems, (iii) human diseases/pathogens, and (iv) cellular processes.

### 3.4. Unique DEGs in B. tabaci MEAM1 and MED Adults

**Figure 4:**
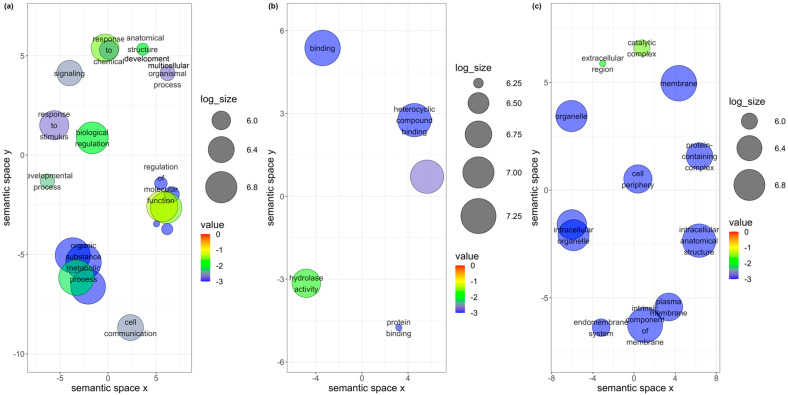
Scatterplots showing (**a**) biological process, (**b**) molecular function, and (**c**) cellular component gene ontology terms for *Bemisia tabaci* Middle East-Asia Minor 1 following the acquisition of viruses. Cluster representatives in a two-dimensional space were derived by applying multidimensional scaling to a matrix of the gene ontology terms’ semantic similarities. Bubble color indicates the *p*-value, and size indicates the frequency of the GO term in the underlying GOA database.

Of the 881 DEGs found in the *B. tabaci* MEAM1 adults, 735 were found to be uniquely and differentially expressed following the virus acquisition (Figure 1a). Using the functional analysis tools available in the OmicsBox version 1.4.11, 526 of the 735 genes were assigned functional groups under three classification systems: biological process (290 genes), molecular function (387 genes), and cellular component (296 genes). Twenty-two GO terms were assigned under the biological process category, of which 20 terms were significant (Figure 4a). Ten GO terms were assigned under the molecular function category, five (binding, protein binding, hydrolase activity, heterocyclic compound binding, and organic cyclic compound binding) of which were significant (Figure 4b). In the cellular component category, sixteen GO terms were identified, and 12 of those were significant (Figure 4c).

**Figure 5:**
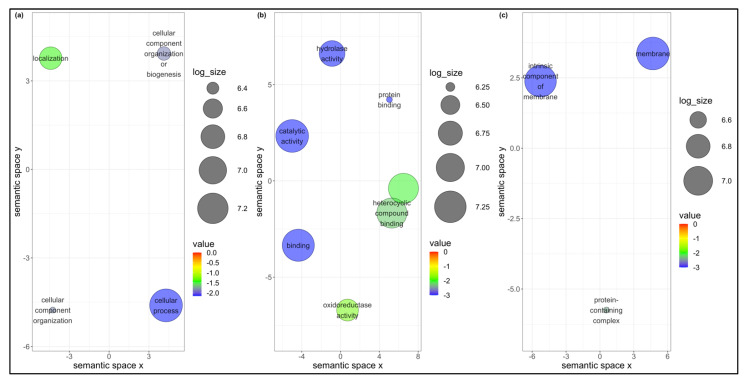
Scatterplots showing (**a**) biological process, (**b**) molecular function, and (**c**) cellular component gene ontology terms for *Bemisia tabaci* Mediterranean following the acquisition of viruses. Cluster representatives in a two-dimensional space were derived by applying multidimensional scaling to a matrix of the gene ontology terms’ semantic similarities. Bubble color indicates the *p*-value, and size indicates the frequency of the GO term in the underlying GOA database.

Of the 559 DEGs found in *B. tabaci* MED adults, 413 were found to be uniquely and differentially expressed following the virus acquisition (Figure 1a). Using functional analysis tools, 223 genes of the 413 genes were assigned functional groups under three classification systems: biological process (120 genes), molecular function (179 genes), and cellular component (107 genes). Sixteen GO terms were assigned under the biological process category, of which four (biogenesis, cellular process, cellular component organization, and localization) were significant (Figure 5a). Eight GO terms were assigned under the molecular function category, of which seven were significant (Figure 5b). In the cellular component category, eleven GO terms were identified, of which three (membrane, intrinsic component of membrane, and protein-containing complex) were significant (Figure 5c).

Only 44.8% (329 of 735) of the total number of DEGs in the *B. tabaci* MEAM1 adults were annotated using the KEGG analysis [63] and were mapped to 301 biochemical pathways (Table S4c,d). The most represented pathway categories were (i) metabolism, (ii) human diseases, and (iii) organismal systems. For the *B. tabaci* MED adults, 42.4% (175 of 413) of the total number of DEGs were annotated using the KEGG analysis [63] and were mapped to 162 biochemical pathways (Table S4e,f). Similar to the MEAM1 whiteflies, metabolism, human diseases/pathogens, and organismal systems were the most represented pathway categories.

### 3.5. Co-Expression Networks from B. tabaci MEAM1 and MED Adults

**Figure 6:**
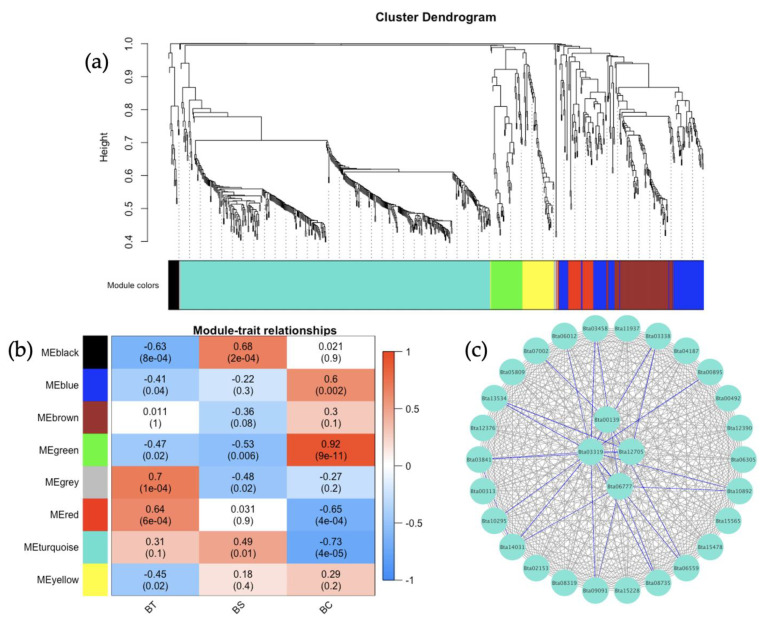
*Bemisia tabaci* Middle East-Asia Minor 1 (MEAM1) adults weighted gene co-expression network analysis. (**a**) Dendrogram clustering showing eight modules of co-expressed genes. A total of 784 genes are represented in this network, with 454 genes belonging to MEturquoise. (**b**) Heatmap showing the correlation of module eigengenes in relation to *B*. *tabaci* MEAM1 that acquired TYLCV (BT), SiGMV (BS), or CuLCrV (BC). (**c**) Top 30 genes from MEturquoise with connectivity lines (blue) associated with the top 5% of the connected genes.

**Figure 7:**
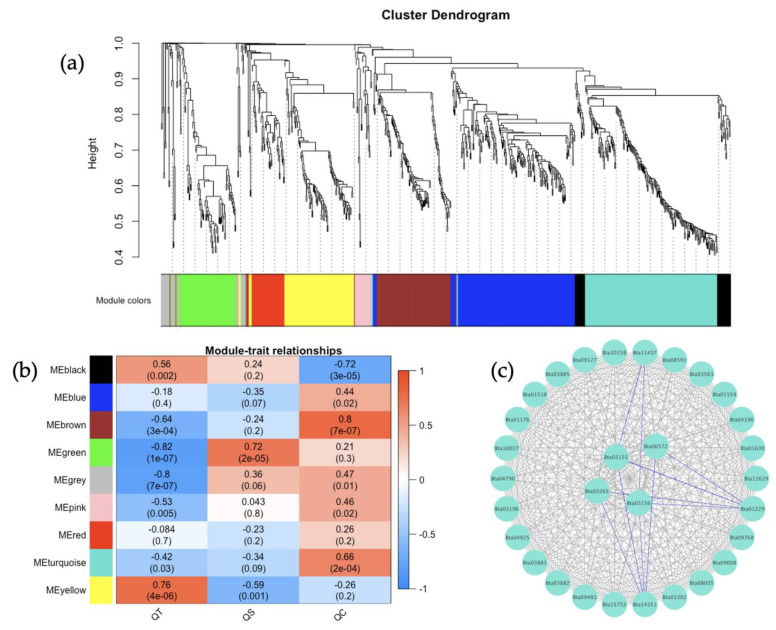
*Bemisia tabaci* Mediterranean (MED) adults weighted gene co-expression network analysis. (**a**) Dendrogram clustering showing nine modules of co-expressed genes. A total of 784 genes are represented in this network, with 188 genes belonging to MEturquoise. (**b**) Heatmap showing the correlation of module eigengenes in relation to *B*. *tabaci* MED that acquired TYLCV (QT), SiGMV (QS), or CuLCrV (QC). (**c**) Top 30 genes from MEturquoise with connectivity lines (blue) associated with the top 5% of the connected genes.

The co-expression of the genes from the *B. tabaci* MEAM1 and MED adults that acquired TYLCV, SiGMV, or CuLCrV were identified in relation to the expression data. Weighted gene co-expression network analysis (WGCNA), which clusters genes into modules based on weighted gene–gene interactions, was used to study the co-expression. For *B. tabaci* MEAM1, eight modules were identified (Figure 6a), and a Pearson’s correlation coefficient analysis showed the connections between the three virus species (Figure 6b). The *B. tabaci* MEAM1 interactions for the largest module, MEturqoiuse, were checked for the top interacting genes among the 30 identified genes (Figure 6c). The top four most highly connected genes were: Bta03319 (Tat-linked quality control protein TatD), Bta00139 (Riboflavin kinase), Bta15228 (Ubiquitin-conjugating enzyme E2 L3, putative), and Bta06777 (GRIP and coiled-coil domain-containing protein, putative). In the *B. tabaci* MED analysis, nine modules were identified (Figure 7a), and a Pearson’s correlation coefficient analysis showed the connections between the three virus species (Figure 7b). The *B. tabaci* MED interactions for the largest module, MEturqoiuse, were checked for the top interacting genes among the 30 identified genes (Figure 7c). The top four most highly connected genes were: Bta03156 (Serine protease 7, isoform), Bta03155 (Serine protease), Bta03265 (Serine protease 7, isoform A), and Bta06572 (unknown protein).

For TYLCV, six modules were identified (Figure S1a), and a Pearson’s correlation coefficient analysis showed the connections between *B. tabaci* MEAM1 and MED (Figure S1b). TYLCV interactions for the largest module, MEturqoiuse, were checked for the top interacting genes among the 30 identified genes (Figure S1c). The top three most highly connected genes were all unknown proteins (Bta14024, Bta04891, and Bta08994). For SiGMV, six modules were identified (Figure S2a), and a Pearson’s correlation coefficient analysis showed the connections between the two whitefly cryptic species (Figure S2b). SiGMV interactions for the largest module, MEturqoiuse, were checked for the top interacting genes among the 30 identified genes (Figure S2c). The top four most highly connected genes were all unknown proteins (Bta10403, Bta01241, and Bta04118), except for Bta13010 (Phosphatidylethanolamine-binding protein). For CuLCrV, five modules were identified (Figure S3a), and a Pearson’s correlation coefficient analysis showed the connections between *B. tabaci* MEAM1 and MED (Figure S3b). CuLCrV interactions for the largest module, MEturqoiuse, were checked for the top interacting genes among the 30 identified genes (Figure S3c). The top three most highly connected genes were: Bta02748 (AP-3 complex subunit mu-1), Bta06159 (Ribosomal protein L19), and Bta14433 (unknown protein).

Following the overview of the functional annotation and pathway analyses of the *B. tabaci* MEAM1 and MED genes, genes that could influence virus–vector interaction were examined.

### 3.6. DEGs among B. tabaci MEAM1 and MED Adults Associated with Virus–Vector Interactions

#### 3.6.1. Virus Infection

Among the common DEGs between *B. tabaci* MEAM1 and MED adults, KEGG annotations revealed three generic biotic stress-induced genes (Table 2) under the “human disease” category. These genes, all underexpressed except in one instance (Table 2), were associated with the infection of two viruses: Human T-cell leukemia virus 1 (Bta03437—Zinc finger protein) and Measles (Bta02903—Heat shock protein 70 and Bta09867—70 kDa heat shock protein) (Table S4b).

In *B. tabaci* MEAM1 adults specifically, 27 genes were associated with different viruses including Human T-cell leukemia virus 1 (four genes), Human immunodeficiency virus 1 (6 genes), Hepatitis B virus (four genes), Hepatitis C virus (one gene), Coronavirus (one gene), Influenza A virus (two genes), Measles virus (three genes), Herpes simplex virus 1 (two genes), Human cytomegalovirus (eight genes), Kaposi sarcoma-associated herpesvirus (three genes), Epstein–Barr virus (three genes), and Human papillomavirus (nine genes) (Table S4d). The majority of the genes (21/27) associated with virus infection were overexpressed, with the Ras-like/related (four genes) and heat shock protein (three genes) genes being the most predominant (Table 2).

Similarly, seven genes, four overexpressed and three underexpressed, were associated with the acquisition of three begomoviruses in *B. tabaci* MED (Table 2). The differentially expressed genes were associated with the infection of Human T-cell leukemia virus 1 (one gene), Human immunodeficiency virus 1 (three genes), Coronavirus (one gene), Measles virus (1 gene), Herpes simplex virus 1 (four genes), Human cytomegalovirus (two genes), and Epstein–Barr virus (two genes) (Table S4f).

#### 3.6.2. Signal Transduction

Among the shared DEGs identified in *B. tabaci* MEAM1 and MED adults, KEGG annotation identified four genes. All but one (Bta02903) were underexpressed (Table 3) and were associated with three signal transduction pathways, namely, AMPK (Bta07645), Apelin (Bta03437), and MAPK (Bta02903 and Bta09867) (Table S4b).

**Figure 8:**
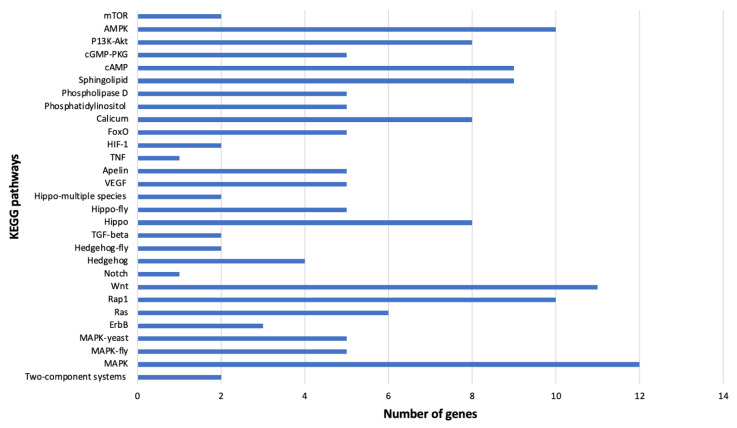
KEGG pathways pertaining to signal transduction identified in viruliferous *Bemisia tabaci* Middle East-Asia Minor 1. The number of differentially expressed genes involved in each pathway are displayed on the *x*-axis.

Fifty-one genes (35 overexpressed and 16 underexpressed) were annotated using the KEGG analysis (Table 3) and associated with 29 signal transduction pathways in MEAM1 whiteflies (Table S4d). The pathways and their associated genes are included below (Figure 8). Some of the prevalent genes in the MEAM1 whiteflies signaling pathways were Ras-like/related genes, protein kinase, acyl-CoS desaturase, heat shock, and guanine nucleotide-binding (Table 3).

**Figure 9:**
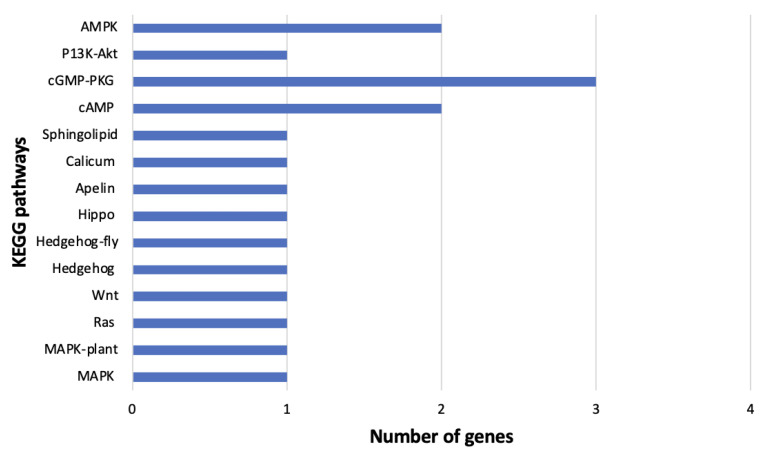
KEGG pathways pertaining to signal transduction identified in viruliferous *Bemisia tabaci* Mediterranean. The number of differentially expressed genes involved in each pathway are displayed on the *x*-axis.

In the *B. tabaci* MED adults, 11 genes (4 overexpressed and 7 underexpressed) were associated with 14 signaling pathways using KEGG annotation (Table 3) (Table S4f). These pathways and the associated genes are included below (Figure 9).

#### 3.6.3. Signaling Molecules and Virus Interaction

Using KEGG annotation, five putative receptor genes (two overexpressed and three underexpressed) were identified in *B. tabaci* MEAM1 adults. They include: Bta03110, Bta01738—Corticotropin-releasing factor receptor 1, Bta15373—Neuropeptide FF receptor 2 under the neuroactive ligand–receptor interaction, Bta04818 under the cytokine–cytokine receptor interaction, and Bta07388—Syndecan under the cell adhesion molecules (Table S4d). The genes Bta03110 and Bta04818 were also associated with both signal transduction and/or virus infection (Table 2 and Table 3). Putative receptor genes were not identified in *B. tabaci* MED adults’ transcriptome data.

#### 3.6.4. Immune Systems

Among the DEGs shared by both *B. tabaci* MEAM1 and MED adults, 10 genes (6 up- and underexpressed depending on whitefly/virus treatment and 4 underexpressed) under the organismal systems were associated with three immune system pathways by KEGG annotation (Table 4). Seven of the ten genes were cathepsin B, while the other three were protein spaetzle and heat shock proteins (Table 4). These genes belonged to the antigen processing and presentation (seven cathepsin B genes) pathway, NOD-like receptor signaling pathway (seven cathepsin B and two heat shock protein genes), and Toll and Imd signaling pathways (protein spaetzle gene) (Table S4b).

A total of 33 genes (21 overexpressed and 12 underexpressed) were annotated using the KEGG analysis (Table 4) and associated with 17 immune system pathways in *B. tabaci* MEAM1 adults (Table S4d). Cathepsin B (seven genes) and Ras-like/related (five genes) genes were the most predominant in this category (Table 4).

In the *B. tabaci* MED adults, 20 genes (6 overexpressed and 14 underexpressed) were associated with six immune systems by KEGG annotation (Table 4). Among the genes identified in the immune systems category, the majority were cathepsins (11 genes), and they belonged to the following pathways: hematopoietic cell lineage (1 genes), platelet activation (2 genes), NOD-like receptor signaling pathway (9 genes), antigen processing and presentation (15 genes), IL-17 signaling pathway (1 gene), and Fc gamma R-mediated phagocytosis (1 gene) (Table S4f).

#### 3.6.5. Cellular Processes (Apoptosis, Lysosome, and Phagosome)

Among the DEGs shared by *B. tabaci* MEAM1 and MED adults, eight cathepsin genes were associated with the apoptosis and lysosome pathways by KEGG annotation (Table S4b). All of those genes were also associated with immune systems, except for Bta20005—cathepsin F (Table 4). The Bta05445-protein transport protein Sec61 subunit β was the only gene associated with phagosome among the shared DEGs identified in *B. tabaci* MEAM1 (fold change: −0.93, treatment: SiGMV) and MED (fold change: 0.89, treatment: TYLCV) adults.

A total of 34 genes (17 overexpressed and 17 underexpressed) were annotated by KEGG analysis (Table 5) and associated with cellular processes in *B. tabaci* MEAM1 adults (Table S4d). Cathepsin F/F-like (nine genes) and cathepsin B (seven genes) genes were the most predominant in this category (Table 5), whereas, among the apoptosis, lysosome, and phagosome pathways, 22, 23, and 7 genes were identified, respectively.

In *B. tabaci* MED adults, 19 genes (6 overexpressed, 12 underexpressed, one in both directions) were associated with cellular processes by KEGG annotation (Table 5). Except for six genes, all were cathepsins (13 genes). The genes in the lysosome (17 genes) and apoptosis (13 genes) pathways were more represented than the genes associated with the phagosome category (four genes) (Table S4f).

### 3.7. DEGs among B. tabaci MEAM1 and MED Adults Associated with Fitness

#### 3.7.1. Reproduction

Using GO annotation, three genes associated with reproduction were identified in *B. tabaci* MEAM1 adults. Bta01855—DNA polymerase ε subunit 3 (fold change: 1.73) and Bta06648—gametocyte-specific factor 1 (fold change: 1.16) were both overexpressed in *B. tabaci* MEAM1 that acquired TYLCV, while Bta01607—ERC protein 2 (fold change: −2.17) was underexpressed in *B. tabaci* MEAM1 that acquired TYLCV. Further, egg production-associated gene Bta07852—vitellogenin (fold change: −1.63) was underexpressed in *B. tabaci* MEAM1 that acquired TYLCV. In both *B. tabaci* MEAM1 and MED adults, another vitellogenin gene (Bta11903) was overexpressed (fold change: 1.61) in *B. tabaci* MEAM1 adults that acquired CuLCrV, while it was underexpressed (fold change: −1.32) in *B. tabaci* MED adults that acquired TYLCV.

#### 3.7.2. Longevity

Based on the KEGG pathway annotation, three genes (two overexpressed/underexpressed and one underexpressed) were associated with two aging pathways among the DEGs shared between *B. tabaci* MEAM1 and MED adults. These pathways included longevity regulating pathway—worm (Bta14177—fatty acyl-CoA reductase 1) and longevity regulating pathway—multiple species (Bta02903 and Bta09867) (Table S4b). The Bta02903 and Bta09867 genes were associated with several pathways (Table 2, Table 3 and Table 4). Bta14177 was overexpressed (fold change: 1.59) in *B. tabaci* MEAM1 adults that acquired SiGMV, while it was underexpressed (fold change: −1.93) in *B. tabaci* MED adults that acquired TYLCV.

Fifteen genes (five overexpressed and ten underexpressed) were annotated using the KEGG analysis (Table 6) and associated with aging in *B. tabaci* MEAM1 adults (Table S4d). Acyl-CoA related (five genes) and heat shock protein (four genes) genes were the most predominant in this category (Table 6). Three, nine, and four genes were classified under longevity regulating pathway, longevity regulating pathway—worm, and longevity regulating pathway—multiple species, respectively.

In *B. tabaci* MED adults, five genes (two overexpressed and three underexpressed) were identified in the aging pathways based on KEGG annotation (Table 6). Four acyl-CoA-related genes and the heat shock protein gene were grouped under longevity regulating pathway—worm and longevity regulating pathway—multiple species, respectively (Table S4f).

## 4. Discussion

This study assessed the differences in gene expression in *B. tabaci* MEAM1 and MED whiteflies in response to the acquisition of Old and New World begomoviruses. Both the Old and New World viruses examined in this study were transmitted by the *B. tabaci* MEAM1 cryptic species, and only the Old World virus (TYLCV) was transmitted by the *B. tabaci* MED cryptic species [36]. Gene expression differences were compared between the two cryptic species following the acquisition of TYLCV, SiGMV, or CuLCrV. Across all three virus treatments, more transcriptional changes were recorded with *B. tabaci* MEAM1 adults (881 genes) than with *B. tabaci* MED adults (559 genes), which represented only 5.6% and 3.6%, respectively, of the overall number of genes (15,668) reported for the *B. tabaci* MEAM1 genome (http://www.whiteflygenomics.org/cgi-bin/bta/index.cgi accessed on 15 December 2021). The high number of transcriptional responses occurring in *B. tabaci* MEAM1 whitefly as opposed to the *B. tabaci* MED whiteflies could be due to the reduced interactions of the two New World begomoviruses in *B. tabaci* MED adults.

The findings indicated that begomovirus species induced variable transcriptional changes in whiteflies upon acquisition. In agreement, previous studies have also reported variable transcriptional responses in whiteflies upon virus acquisition. For instance, 236 genes were differentially expressed in *B. tabaci* MEAM1 adults that acquired tomato yellow leaf curl China virus (TYLCCNV) compared with non-viruliferous whiteflies [71]. Two other studies using the same whitefly cryptic species and virus species reported different numbers of differentially expressed genes in *B. tabaci* MEAM1 that acquired TYLCCNV when compared with their non-viruliferous counterparts. There were 457 genes in the study of Luan et al. [50], whereas there were 1606 genes in the study of Luan et al. [44]. Similarly, in the TYLCV pathosystem, two independent studies reported a contrasting number of differentially expressed genes (79 genes versus 1347 genes) in viruliferous *B. tabaci* MEAM1 compared with non-viruliferous, while another study identified 78 differentially expressed genes in viruliferous *B. tabaci* MED, compared with the non-viruliferous treatment [47,48,49]. Further, contrasting transcriptional changes in the whitefly have been observed in semi-persistently transmitted criniviruses. For example, for the tomato chlorosis virus (ToCV) pathosystem, one study reported 221 differentially expressed genes in viruliferous *B. tabaci* MED adults compared with non-viruliferous adults, whereas another study reported 1155 differentially expressed genes in viruliferous *B. tabaci* MEAM1 adults compared with non-viruliferous adults [45,49]. In another crinivirus pathosystem, cucurbit yellow stunting disorder virus, 262 differentially expressed genes were reported in viruliferous *B. tabaci* MEAM1 compared with non-viruliferous counterparts [46]. The differences in the experimental design and analysis, such as: (i) AAP from 1–7 days, (ii) viruliferous whiteflies with no gut clearing to minimize potential indirect host plant effects, (iii) low throughput compared with high throughput sequencing platforms, (iv) the different number of libraries sequenced, and (v) bioinformatics analyses, could have partly contributed to the observed variations in the number of transcriptional changes observed in whiteflies in different studies. 

This study investigated the transcriptional changes in *B. tabaci* MEAM1 and MED induced by three plant viruses belonging to the same genus, *Begomovirus*, and differential gene expression patterns varied between them. More transcriptional changes were associated with TYLCV acquisition (MEAM1–462 genes and MED–413 genes), followed by SiGMV acquisition (MEAM1–459 genes and MED–165 genes), whereas CuLCrV acquisition (MEAM1–69 genes and MED–44 gene) induced the least number of transcriptional changes. Both TYLCV and SiGMV accumulated at significantly higher levels (copies per ng DNA) in both their host plants and vector’s tissues (midgut, hemolymph, and salivary glands), while CuLCrV accumulated at significantly lower amounts in its host plant and vector’s tissues [30,36]. *Bemisia tabaci* MEAM1 transmits SiGMV and CuLCrV, and their circulative tropism in whiteflies could have facilitated binding with different whitefly proteins and putative receptors, leading to increased transcriptional changes [22,72,73]. *Bemisia tabaci* MED did not transmit the two New World viruses and accumulated at reduced amounts in its tissues; hence, the reduced circulative tropism of New World viruses could have influenced the lower transcriptional responses compared to TYLCV. The differences in *B. tabaci* MEAM1 and MED in terms of acquisition and the competency to inoculate different begomoviruses observed here and in previous studies [74,75] support previous hypotheses surrounding *B*. *tabaci*-begomovirus transmission specificity.

The acquisition of three begomoviruses by *B. tabaci* MEAM1 and MED resulted in transcriptional changes, of which 146 genes were differentially expressed in common between the two invasive *B. tabaci* species. KEGG analysis annotated only 42 of the 146 genes, and the most represented pathways were classified under metabolic pathways involving carbohydrate and lipid metabolism. Virus (hepatitis C virus and HIV) infection and replication have been reported to increase carbohydrate and lipid metabolism in infected CD4^+^ T cells [76,77]. The majority of the genes associated with lipid metabolism in both *B. tabaci* MEAM1 and MED were underexpressed, and those associated with carbohydrate metabolism were underexpressed in *B. tabaci* MEAM1 and partly overexpressed in *B. tabaci* MED. The results herein are consistent with previous reports of the downregulation of genes associated with lipid metabolism in *B. tabaci* MEAM1 or MED post acquisition of ToCV, TYLCCNV, and TYLCV, or of TYLCV and ToCV from co-infected plants [44,49]. In contrast, some studies have reported the upregulation of genes associated with lipid metabolism in a circulative and propagative virus study system (tomato spotted wilt orthotospovirus-thrips) [78,79]. Evidence for reduced gene expression associated with carbohydrate and lipid metabolism in viruliferous *B. tabaci* MEAM1 and MED may offer another line of evidence to counter the hypothesis that begomoviruses replicate in their whitefly vector [80,81]. Nevertheless, the temporary replication of at least one begomovirus (TYLCV) in *B. tabaci* MEAM1 salivary glands has been reported in a recent study [82]. The replication affected TYLCV accumulation in the salivary glands minimally given the overall virus accumulation within whiteflies.

The weighted gene correlation network analysis (WGCNA) has been applied to analyze various biological processes for different organisms [83,84]. In this study, the gene co-expression network analysis of *B. tabaci* MEAM1 and MED adults that acquired TYLCV, SiGMV, or CuLCrV identified several candidate genes for studying the vector competence of two invasive whiteflies. WGCNA provided scale-free networks between genes within modules, and these modules aided in identifying genes that make *B. tabaci* MEAM1 a more competent vector of New World begomoviruses (SiGMV and CuLCrV) than its sister cryptic species *B. tabaci* MED. For instance, among the top four interacting genes identified in the largest module for *B. tabaci* MEAM1 was the gene *Bta15228* (ubiquitin-conjugating enzyme E2 L3, putative). The ubiquitin-conjugating enzyme was required for Notch signaling activation during *Drosophila* wing development. In addition, this gene was involved in the endocytic trafficking of the Notch protein [85]. This gene could also be involved in the endocytic trafficking of virus particles in *B. tabaci* MEAM1. In *B. tabaci* MED, three serine protease genes (*Bta03155*, *Bta03156*, and *Bta03265*) were identified in the largest module among the top four interacting genes. Serine proteases are proteolytic enzymes responsible for digestion, larval-pupal molting, signal transduction, and immune responses in insects [86,87,88,89]. The identification of serine protease genes among the top interacting genes in *B. tabaci* MED adults could be signatures of heightened innate immune responses such as melanization and the production of antimicrobial peptides triggered by the acquisition of begomoviruses. The ubiquitin-conjugating enzyme and serine protease genes identified in *B. tabaci* MEAM1 and MED adults, respectively, could be important targets for future investigation.

Similarly, the gene co-expression network analysis for TYLCV, SiGMV, or CuLCrV acquired by *B. tabaci* MEAM1 and MED adults was examined with the purpose of identifying candidate genes involved in the acquisition of Old and New World begomoviruses. None of the top three interacting genes identified in the largest module for TYLCV, an Old World begomovirus, were functionally annotated. In contrast, a majority of the top three/four interacting genes identified in the largest module for New World begomoviruses—namely, SiGMV and CuLCrV—were functionally annotated. For instance, among the top four interacting genes identified in the largest module for SiGMV was the gene *Bta13010*—phosphatidylethanolamine-binding protein (PEBP). The downregulation of PEBP genes in the *Bombyx mori* strain resistant to *Bombyx mori* nucleopolyhedrovirus (BmNPV) upon infection induced enhanced apoptosis, thereby repressing the ability of BmNPV to infect other cells [90]. In *B. tabaci* MED adults that transmitted TYLCV, the relative expression of PEBP4 was increased significantly and was shown to interact with the TYLCV coat protein, putatively influencing apoptosis and autophagy mechanisms in the whitefly [91]. The potential for the direct role of PEBP4 in *B. tabaci* MEAM1 capable of transmitting SiGMV remains to be investigated; however, based on the increased relative expression observed in this study and the prior inference, PEBP4 may be conferring a similar function across multiple begomovirus-vector systems. Among the three interacting genes identified in the largest module for CuLCrV was the gene *Bta02748* (AP-3 complex subunit mu-1). Adaptin proteins (AP) are membrane-bound heterotetrameric complexes localized in cellular buds and vesicles, and their main role is intracellular trafficking in the trans-Golgi network [92]. The interaction of AP with the human respiratory syncytial virus matrix protein was essential for the latter’s trafficking in the host cells [93]. The regulation of the AP-3 complex subunit mu-1 in the whiteflies that acquired a New World begomovirus could play a role in virus tropism, although this requires functional validation.

KEGG analysis identified many genes implicated in human virus infection that were differentially regulated in viruliferous *B. tabaci* MEAM1 (21 overexpressed and 9 underexpressed genes) and MED (5 overexpressed and 5 underexpressed genes) adults compared with non-viruliferous counterparts. For example, in *B. tabaci* MEAM1 adults that acquired SiGMV, *Bta14253*—protein kinase C was overexpressed compared with non-viruliferous adults. This gene was implicated in the infection of human immunodeficiency virus 1 (HIV), hepatitis B virus, coronavirus, influenza A virus, and human cytomegalovirus by KEGG analysis. Protein kinase C agonists are reported to induce latent HIV expression from viral reservoirs and protect primary CD4^+^ T cells from HIV infection through the down-modulation of HIV coreceptor expression [94]. Another class of genes, zinc finger proteins (*Bta01535* and *Bta03437*) was underexpressed in *B. tabaci* MEAM1 adults that acquired TYLCV compared with their non-viruliferous counterparts. In addition, these genes (zinc finger proteins) were also modulated in both directions (up and down) in *B. tabaci* MED adults that acquired SiGMV compared with their non-viruliferous counterparts. Zinc finger proteins were also implicated in herpes simplex virus 1 infection (HSV-1). The upregulation of zinc finger proteins was reported to play a role in HSV-1 replication and binding to promoter proteins [95]. Five heat shock proteins (*Bta00008*, *Bta02903*, *Bta03000*, *Bta06076*, and *Bta14532*) were all, except in one instance, underexpressed in viruliferous whiteflies compared with non-viruliferous counterparts. The increased expression of heat shock proteins was reported after measles virus infection, resulting in increased cytopathic effects [96]. The role of the above-selected genes in both *B. tabaci* MEAM1 and MED adults following the acquisition of begomoviruses is unknown and warrants further investigation.

For the successful circulative translocation of begomoviruses in whiteflies, the viral coat protein must interact with putative receptors at the midgut, in the hemolymph, and at the primary salivary glands [22]. A few putative receptors such as the GroEL chaperone protein, heat shock proteins, midgut proteins, peptidyl-prolyl isomerase protein, and peptidoglycan recognition protein gene were identified in previous studies [23,24,25,26,27]. In addition to those, this study identified other possible putative receptors in the cytokine–cytokine receptor interaction and cell adhesion molecule pathways that may play a role in the circulative movement of begomoviruses in their vectors, and they include *Bta04818*—type I serine/threonine kinase receptor and *Bta07388*—syndecan. Serine/threonine kinase, an anchored protein-associated kinase in mammalian cells, was reported as an important cellular component in regulating the entry or clathrin-mediated endocytosis of rabies virus (RABV) [97]. Syndecan is a cell surface heparan sulphate proteoglycan (HSPGs), which plays several roles including virus (hepatitis E virus and herpes simplex virus) attachment and entry [98]. Hepatitis E virus, human papillomavirus, and HIV are some of the viruses reported to bind to cell surface HSPGs for their initial attachment to host cells [99,100,101]. The upregulation of the type I serine/threonine kinase receptor and syndecan genes in *B. tabaci* MEAM1 adults that acquired SiGMV could be associated with their binding to viral DNA, thereby enabling circulative translocation. Aminopeptidase N is another gene that was differentially expressed (underexpressed) in both *B. tabaci* MEAM1 and MED adults that acquired TYLCV. This gene, a plant virus receptor, was overexpressed in aphids and thrips that acquired pea enation mosaic virus and tomato spotted wilt virus (TSWV), respectively [78,102]. Whether the aminopeptidase N gene plays any role in begomovirus (TYLCV) reception in either *B. tabaci* MEAM1 or MED adults is unknown at this juncture.

Whiteflies have an innate immune system that has been documented against pathogens [103,104]. The RNA interference (RNAi) pathway is the major mechanism insects use against virus infection [43]. In addition, other innate antimicrobial pathways such as Imd, Toll, JAK-STAT, phagocytosis, apoptosis, and proteolysis were reported to play crucial roles in insects’ (Diptera and Lepidoptera) antiviral responses [105,106,107]. A majority of the genes associated with innate immune pathways in the viruliferous *B. tabaci* MEAM1 adults were overexpressed, as opposed to the viruliferous *B. tabaci* MED adults, where they were underexpressed (Table 4 and Table 5). For instance, several cathepsin B genes were identified in both *B. tabaci* MEAM1 and MED adults. These genes were associated with apoptosis and immune system pathways such as the NOD-like receptor signaling pathway and antigen processing and presentation pathway. Further, cathepsins are implicated to play a role in virus transmission [108,109]. All of the nine genes identified in *B. tabaci* MED were underexpressed, while four genes were overexpressed and three were underexpressed in *B. tabaci* MEAM1 adults (Table 4). Similar to our findings, several studies have identified the differential expression (both up- and downregulation) of genes such as cathepsins associated with innate immune pathways in whiteflies following begomovirus or crinivirus acquisition [44,45,46,47,49,51]. The contrasting differential expression of genes under the innate immune pathways such as cathepsins in *B. tabaci* MEAM1 and MED identified in this study possibly highlights differences in the immune responses to virus acquisition or other vector-virus interactions between the two *B. tabaci* cryptic species. Ras-like GTP-binding proteins were other immune response genes identified only in *B. tabaci* MEAM1 adults that acquired TYLCV or SIGMV. Like *B. tabaci* MEAM1 adults that acquired TYLCV or SiGMV, ras-like GTP-binding proteins were overexpressed in thrips that were exposed to TSWV [78]. The upregulation of these genes in viruliferous *B. tabaci* MEAM1 adults could be another mechanism evolved by whiteflies to counter begomoviruses.

The exposure to begomoviruses, especially TYLCV and CuLCrV, affected the expression of several fitness-related genes in *B. tabaci* MEAM1 and MED. This study identified the up- and downregulation of genes associated with egg production, spermatogenesis, and longevity in whiteflies that acquired begomoviruses. For example, *Bta06648*—gametocyte-specific factor 1 (GTSF1) gene was overexpressed in *B. tabaci* MEAM1 adults that acquired TYLCV. In *Drosophila*, this gene was essential for P-element-induced wimpy testis-interacting RNA-mediated transcriptional repression, the histone mediated repression of transposons, and their neighboring genes in the ovary [110]. Similarly, in mice, this gene was essential for spermatogenesis and transposon suppression in mouse testes [111]. The upregulation of the GTSF1 gene in *B. tabaci* MEAM1 adults that acquired TYLCV may increase the development of sperm cells in their male reproductive organs, thereby influencing reproduction. Two egg production associated genes—vitellogenin (*Bta07852* and *Bta11903*) were underexpressed in *B. tabaci* MEAM1 and MED adults that acquired TYLCV, whereas one vitellogenin gene (*Bta11903*) was overexpressed in *B. tabaci* MEAM1 adults that acquired CuLCrV. Further, most genes associated with aging pathways in viruliferous *B. tabaci* MEAM1 and MED adults were underexpressed. Positive, neutral, and negative effects of begomovirus infection on whitefly fecundity and longevity have been reported [42,112,113,114]. Evidence at the transcriptome level for the underexpression of the egg production gene in *B. tabaci* MEAM1 and MED adults that acquired TYLCV, as indicated in this study, is consistent with the Pan et al. [114] study. That study reported that *B. tabaci* MEAM1 and MED that acquired TYLCV produced fewer eggs on cotton than their non-viruliferous counterparts [114]. In contrast, the overexpression of oviposition-related genes in *B. tabaci* MEAM1 that acquired CuLCrV may indicate higher fecundity, but this assumption was not supported by Gautam et al. [42]. There were no significant differences in the fecundity of *B. tabaci* MEAM1 females that acquired CuLCrV and their non-viruliferous counterparts [42]. Gautam et al. [36] demonstrated that the fitness effects of both *B. tabaci* MEAM1 and MED did not vary within each host species, and in this study, host effects were eliminated by gut clearing for 72 h. Consequently, this study predominantly provides evidence at the transcriptome level for some of the begomoviruses-induced (predominantly direct) macro-level fitness effects on their vectors—*B. tabaci* MEAM1 and MED.

## 5. Conclusions

The acquisition of begomoviruses by two invasive whiteflies induced variable transcriptional responses. Increased transcriptional changes were observed in *B. tabaci* MEAM1 adults compared to *B. tabaci* MED adults. Although the three plant viruses belong to the same genus, *Begomovirus*, the gene expression patterns varied between them. The gene co-expression network analysis for *B. tabaci* MEAM1 and MED that acquired begomoviruses revealed some candidate genes such as the ubiquitin-conjugating enzyme, which could be involved in the endocytic trafficking of virus particles. In addition, other possible putative receptors such as type I serine/threonine kinase receptor and syndecan were identified, and these could be involved in the circulative movement of begomoviruses in their vectors. Several genes implicated in the infection of human viruses were differentially regulated in viruliferous whiteflies. Furthermore, under the innate immune pathways, the contrasting differential expression of genes such as cathepsins occurred between *B. tabaci* MEAM1 and MED adults that acquired the begomoviruses. The differences in the immune responses of *B. tabaci* MEAM1 and MED adults upon virus acquisition possibly highlight their unique defense mechanisms against begomoviruses. Multiple genes were identified that may play a role in the New World begomovirus transmission. These could be important targets to investigate in the future. This study also provides evidence at the transcriptome level for some of the observed macro-level fitness effects of begomoviruses on their whitefly vectors.

## Figures and Tables

**Table 1:** Summary of RNA sequencing datasets generated from *Bemisia tabaci* MEAM1 and MED adults provided with a feeding access for 72 h on tomato yellow leaf curl virus-infected or non-infected tomato plants, sida golden mosaic virus-infected or non-infected prickly sida plants, and cucurbit leaf crumple virus-infected or non-infected squash plants.

Whitefly (wf)	Sample Description	No. Raw Read Pairs	No. Final Cleaned Read Pairs	Mapped to *B. tabaci* MEAM1 Genome	
				No. Mapped	% Mapped
MEAM1	TYLCV viruliferous wf rep 1	27,270,100	25,637,467	24,019,254	93.69
	TYLCV viruliferous wf rep 2	33,377,647	31,071,277	29,119,197	93.72
	TYLCV viruliferous wf rep 3	24,223,840	21,083,268	19,512,995	92.55
	TYLCV viruliferous wf rep 4	22,603,100	20,914,166	19,565,243	93.55
	TYLCV viruliferous wf rep 5	27,080,141	25,645,471	23,601,484	92.03
	TYLCV non-viruliferous wf rep 1	23,986,058	20,725,007	19,011,846	91.73
	TYLCV non-viruliferous wf rep 2	22,543,749	21,484,751	20,078,449	93.45
	TYLCV non-viruliferous wf rep 3	26,130,542	25,088,690	23,641,100	94.23
	TYLCV non-viruliferous wf rep 4	19,634,831	18,563,885	16,658,491	89.74
	SiGMV viruliferous wf rep 1	24,481,178	22,306,202	20,999,224	94.14
	SiGMV viruliferous wf rep 2	25,457,659	25,032,825	23,802,144	95.08
	SiGMV viruliferous wf rep 3	29,098,951	26,525,917	24,902,267	93.88
	SiGMV non-viruliferous wf rep 1	24,332,531	24,082,074	22,848,884	94.88
	SiGMV non-viruliferous wf rep 2	25,933,031	25,672,553	24,281,335	94.58
	SiGMV non-viruliferous wf rep 3	22,759,943	22,505,467	21,186,253	94.14
	CuLCrV viruliferous wf rep 1	21,397,610	19,723,901	18,448,967	93.54
	CuLCrV viruliferous wf rep 2	22,301,938	20,532,734	19,168,732	93.36
	CuLCrV viruliferous wf rep 3	21,582,185	19,996,867	18,700,091	93.52
	CuLCrV viruliferous wf rep 4	21,650,343	20,031,545	18,739,720	93.55
	CuLCrV viruliferous wf rep 5	19,784,311	18,353,651	17,045,909	92.87
	CuLCrV non-viruliferous wf rep 1	23,300,250	21,146,623	19,768,756	93.48
	CuLCrV non-viruliferous wf rep 2	22,140,101	20,294,419	19,013,129	93.69
	CuLCrV non-viruliferous wf rep 3	22,523,401	20,735,034	19,428,624	93.70
	CuLCrV non-viruliferous wf rep 4	21,194,688	19,576,275	18,327,411	93.62
	CuLCrV non-viruliferous wf rep 5	22,698,997	20,790,783	19,433,502	93.47
MED	TYLCV viruliferous wf rep 1	23,977,947	21,535,882	19,304,336	89.64
	TYLCV viruliferous wf rep 2	24,395,357	21,999,676	19,854,074	90.25
	TYLCV viruliferous wf rep 3	20,9762,11	18,879,921	17,061,784	90.37
	TYLCV viruliferous wf rep 4	26,209,320	23,610,513	21,313,232	90.27
	TYLCV non-viruliferous wf rep 1	20,685,800	18,272,491	16,550,563	90.58
	TYLCV non-viruliferous wf rep 2	22,763,950	20,476,851	18,633,954	91.00
	TYLCV non-viruliferous wf rep 3	23,892,860	21,062,128	19,086,844	90.62
	TYLCV non-viruliferous wf rep 4	22,570,010	19,970,201	18,086,769	90.57
	TYLCV non-viruliferous wf rep 5	20,134,315	17,960,386	16,234,631	90.39
	SiGMV viruliferous wf rep 1	22,732,648	20,756,619	18,734,819	90.26
	SiGMV viruliferous wf rep 2	19,747,880	18,122,103	16,407,875	90.54
	SiGMV viruliferous wf rep 3	22,988,951	20,864,408	18,730,928	89.77
	SiGMV viruliferous wf rep 4	22,521,272	20,582,499	18,583,828	90.29
	SiGMV viruliferous wf rep 5	29,236,819	26,690,404	24,037,689	90.06
	SiGMV non-viruliferous wf rep 1	26,310,239	24,295,385	21,962,427	90.40
	SiGMV non-viruliferous wf rep 2	23,960,072	22,029,656	19,493,192	88.49
	SiGMV non-viruliferous wf rep 3	24,484,549	22,676,536	20,522,495	90.50
	SiGMV non-viruliferous wf rep 4	22,282,789	20,521,023	18,454,698	89.93
	SiGMV non-viruliferous wf rep 5	29,952,155	27,649,370	25,014,401	90.47
	CuLCrV viruliferous wf rep 1	22,647,116	19,812,026	17,824,979	89.97
	CuLCrV viruliferous wf rep 2	22,271,955	19,857,105	17,933,941	90.31
	CuLCrV viruliferous wf rep 3	21,412,367	17,573,543	15,635,887	88.97
	CuLCrV viruliferous wf rep 4	21,739,525	19,470,833	17,583,080	90.30
	CuLCrV viruliferous wf rep 5	22,185,444	18,994,180	16,993,665	89.47
	CuLCrV non-viruliferous wf rep 1	21,346,943	18,119,382	16,207,549	89.45
	CuLCrV non-viruliferous wf rep 2	21,931,996	18,517,714	16,503,842	89.12
	CuLCrV non-viruliferous wf rep 3	28,625,279	24,307,191	21,703,554	89.29

Note. After the 72 h feeding access period, the whiteflies were transferred to cotton plants for another 72 h for gut clearing.

**Table 2:** Differential expression of genes associated with virus infection in viruliferous *Bemisia tabaci* Middle East-Asia Minor 1 (MEAM1) and Mediterranean (MED) adults compared with non-viruliferous adults.

Gene ID	Annotation	FC in Whitefly & Virus Treatment
DEGs shared by MEAM1 and MED		
*Bta03437*	Zinc finger protein	−2.69 for B-TYLCV, −1.38 for Q-SiGMV
*Bta02903*	Heat shock protein 70	−5.55 for B-CuLCrV, 1.83 for Q-CuLCrV
*Bta09867*	70 kDa heat shock protein	−4.54 for B-CuLCrV, −1.50 for Q- TYLCV, −2.63 for Q-SiGMV
DEGs specific to MEAM1		
*Bta00981*	Cyclic AMP-responsive element-binding protein 3-like protein 2	1.60 for SiGMV
*Bta03000*	Heat shock protein 70	−5.91 for CuLCrV
*Bta03004*	Suppressor of hairless protein	1.07 for TYLCV, 1.29 for SiGMV
*Bta03628*	Zinc finger protein 569	−1.30 for SiGMV
*Bta04025*	Serine/threonine-protein phosphatase 2A regulatory subunit B subunit beta	1.02 for SiGMV
*Bta04026*	Serine/threonine-protein phosphatase 2A regulatory subunit B subunit beta	−1.67 for TYLCV
*Bta04167*	Ras-like GTP-binding protein RHO	1.22 for SiGMV
*Bta04484*	Guanine nucleotide-binding protein subunit alpha-like protein	1.53 for TYLCV
*Bta04715*	Ras-related C3 botulinum toxin substrate 1	1.69 for TYLCV, 1.49 for SiGMV
*Bta04818*	Type I serine/threonine kinase receptor	1.47 for SiGMV
*Bta06076*	Heat shock protein	−1.39 for TYLCV
*Bta06234*	Guanine nucleotide-binding protein G(O) subunit alpha	1.34 for SiGMV
*Bta08159*	Transcription factor 7-like 1-A	1.50 for SiGMV
*Bta10215*	Ran-specific GTPase-activating protein	1.29 for TYLCV
*Bta10683*	AP-1 complex subunit sigma-2	1.56 for TYLCV, 1.59 for SiGMV
*Bta11558*	Ras-related C3 botulinum toxin substrate 1	1.22 for TYLCV, 1.29 for SiGMV
*Bta11785*	Zinc finger protein	0.86 for CuLCrV
*Bta12195*	Phosphoinositide phospholipase C	1.15 for SiGMV
*Bta13153*	Sensory neuron membrane protein 1	1.04 for SiGMV
*Bta13437*	Actin	−1.84 for TYLCV
*Bta14253*	Protein kinase C	1.54 for SiGMV
*Bta14532*	70 kDa heat shock protein	−2.20 for CuLCrV
*Bta14568*	Guanine nucleotide-binding protein G(S) subunit alpha	1.12 for TYLCV
*Bta14627*	Mothers against decapentaplegic homolog	1.15 for SiGMV
*Bta15240*	F-box/WD repeat-containing protein 1A	1.19 for SiGMV
*Bta15478*	Ras-like GTP-binding protein RHO	1.45 for SiGMV
*Bta15565*	Partitioning defective protein 6	1.37 for SiGMV
DEGs specific to MED		
*Bta00008*	70 kDa heat shock protein	−2.02 for SiGMV
*Bta00607*	SAM domain and HD domain-containing protein 1	0.71 for TYLCV
*Bta01535*	Zinc finger protein 208	1.28 for SIGMV
*Bta01860*	Calreticulin	0.72 for TYLCV
*Bta02113*	Protein disulfide-isomerase	0.76 for TYLCV
*Bta06159*	Ribosomal protein L19	−3.19 for TYLCV
*Bta12772*	Serine/threonine-protein phosphatase	−0.73 for TYLCV

Note. Genes with the same annotation name but different gene IDs are isoforms. B- and Q- represent the *B. tabaci* MEAM1 and MED.

**Table 3:** Differential expression of genes associated with signal transduction in viruliferous compared with non-viruliferous *Bemisia tabaci* Middle East-Asia Minor 1 and Mediterranean (MED) adults.

Gene ID	Annotation	FC in Whitefly & Virus Treatment
DEGs shared by MEAM1 and MED		
*Bta03437*	Zinc finger protein	−2.69 for B-TYLCV, −1.38 for Q-SiGMV
*Bta02903*	Heat shock protein 70	−5.55 for B-CuLCrV, 1.83 for Q-CuLCrV
*Bta07645*	Prickle, putative	−1.71 for B- TYLCV, −1.26 for Q-TYLCV
*Bta09867*	70 kDa heat shock protein	−4.54 for B-CuLCrV, −1.50 for Q-TYLCV, −2.63 for Q-SiGMV
DEGs specific to MEAM1		
*Bta00915*	Homeobox protein 9	1.29 for SiGMV
*Bta00981*	Cyclic AMP-responsive element-binding protein 3-like protein 2	1.60 for SiGMV
*Bta03000*	Heat shock protein 70	−5.91 for CuLCrV
*Bta03004*	Suppressor of hairless protein	1.07 for TYLCV, 1.29 for SiGMV
*Bta03110*	5-hydroxytryptamine receptor 2A	−1.62 for TYLCV
*Bta03159*	Ras-like protein 2	1.17 for SiGMV
*Bta03261*	Homeobox protein prospero	1.13 for SiGMV
*Bta03841*	Casein kinase	1.11 for SiGMV
*Bta04025*	Serine/threonine-protein phosphatase 2A regulatory subunit B subunit beta	1.02 for SiGMV
*Bta04026*	Serine/threonine-protein phosphatase 2A regulatory subunit B subunit beta	−1.67 for TYLCV
*Bta04098*	Acyl-CoA Z9 desaturase	−1.26 for SiGMV
*Bta04167*	Ras-like GTP-binding protein RHO	1.22 for SiGMV
*Bta04210*	Protein kinase	1.60 for SiGMV
*Bta04351*	Transcriptional enhancer factor TEF-1	−1.62 for TYLCV
*Bta04481*	Calcium release-activated calcium channel protein 1	1.63 for SiGMV
*Bta04484*	Guanine nucleotide-binding protein subunit alpha-like protein	1.53 for TYLCV
*Bta04715*	Ras-related C3 botulinum toxin substrate 1	1.69 for TYLCV, 1.49 for SiGMV
*Bta04818*	Type I serine/threonine kinase receptor	1.47 for SiGMV
*Bta05836*	Protein prickle	−1.79 for TYLCV
*Bta05989*	Inositol hexakisphosphate kinase 2	1.11 for SiGMV
*Bta06076*	Heat shock protein	−1.39 for TYLCV
*Bta06089*	Calcium/calmodulin-dependent protein kinase type II subunit delta	1.51 for TYLCV
*Bta06234*	Guanine nucleotide-binding protein G(O) subunit alpha	1.34 for SiGMV
*Bta07544*	Alkaline phosphatase	−1.86 for TYLCV
*Bta07953*	Raw, isoform A	1.24 for SiGMV
*Bta07992*	Protein kinase C	1.58 for SiGMV
*Bta08159*	Transcription factor 7-like 1-A	1.50 for SiGMV
*Bta08259*	Inositol-trisphosphate 3-kinase B	1.36 for SiGMV
*Bta08332*	Arrestin 1c	−1.89 for TYLCV
*Bta08932*	Acyl-CoA desaturase 1	−1.63 for TYLCV
*Bta08933*	Acyl-CoA desaturase	3.55 for SiGMV
*Bta09047*	Phosphoenolpyruvate carboxykinase [GTP]	−1.25 for TYLCV, −1.21 for SiGMV
*Bta09151*	Protein kinase	−1.51 for TYLCV
*Bta09834*	Sodium/potassium-transporting ATPase subunit beta-2, putative	1.01 for SiGMV
*Bta10421*	E3 ubiquitin-protein ligase CBL, putative	1.22 for SiGMV
*Bta11558*	Ras-related C3 botulinum toxin substrate 1	1.22 for TYLCV, 1.29 for SiGMV
*Bta12096*	Phosphatidate cytidylyltransferase	1.08 for SiGMV
*Bta12195*	Phosphoinositide phospholipase C	1.15 for SiGMV
*Bta13074*	Sodium/calcium exchanger 1	1.84 for SiGMV
*Bta13437*	Actin	−1.84 for TYLCV
*Bta13534*	Ceramide synthase 6	1.28 for SiGMV
*Bta14253*	Protein kinase C	1.54 for SiGMV
*Bta14395*	Alkaline phosphatase	−1.53 for TYLCV, -0.98 for SiGMV
*Bta14493*	E3 ubiquitin-protein ligase	−2.14 for TYLCV
*Bta14532*	70 kDa heat shock protein	−2.20 for CuLCrV
*Bta14568*	Guanine nucleotide-binding protein G(S) subunit alpha	1.12 for TYLCV
*Bta14627*	Mothers against decapentaplegic homolog	1.15 for SiGMV
*Bta15240*	F-box/WD repeat-containing protein 1A	1.19 for SiGMV
*Bta15478*	Ras-like GTP-binding protein RHO	1.45 for SiGMV
*Bta15489*	Transporter	1.08 for SiGMV
*Bta15565*	Partitioning defective protein 6	1.37 for SiGMV
DEGs specific to MED		
*Bta00008*	70 kDa heat shock protein	−2.02 for SiGMV
*Bta01763*	Acyl-CoA Delta(11) desaturase	0.95 for TYLCV
*Bta02612*	Protein kinase C	−0.82 for TYLCV
*Bta04657*	Speckle-type POZ protein B	−0.79 for TYLCV
*Bta05672*	Acyl-CoA desaturase	−1.62 for TYLCV
*Bta09402*	Phospholipase A2	1.81 for TYLCV
*Bta09287*	Chaperone protein HtpG	0.73 for TYLCV
*Bta12301*	Nucleoside diphosphate kinase	0.83 for SiGMV
*Bta12772*	Serine/threonine-protein phosphatase	−0.73 for TYLCV
*Bta14581*	Calcium-transporting ATPase	−1.79 for CuLCrV
*Bta14821*	Wnt inhibitory factor 1	−1.24 for TYLCV

Note. Genes with the same annotation name but different gene IDs are isoforms. B- and Q- represent the *B. tabaci* MEAM1 and MED.

**Table 4:** Differential expression of genes associated with immune system categories in viruliferous compared with non-viruliferous *Bemisia tabaci* Middle East-Asia Minor 1 (MEAM1) and Mediterranean (MED) adults.

Gene ID	Annotation	FC in Whitefly & Virus Treatment
DEGs shared by MEAM1 and MED		
*Bta02886*	Protein spaetzle	1.13 for B-CuLCrV, −1.35 for Q-TYLCV
*Bta02903*	Heat shock protein 70	−5.55 for B-CuLCrV, 1.83 for Q-CuLCrV
*Bta08035*	Cathepsin B	−1.55 for B-TYLCV, −1.19 for Q-TYLCV
*Bta09314*	Cathepsin B	−2.17 for B-SiGMV, 0.89 for Q-TYLCV, 1.18 for Q-SiGMV
*Bta09772*	Cathepsin B	−1.11 for B-SiGMV, 1.02 for Q-TYLCV, −1.67 for Q-SiGMV
*Bta09867*	70 kDa heat shock protein	−4.54 for B-CuLCrV, −1.50 for Q-TYLCV, −2.63 for Q-SiGMV
*Bta10364*	Cathepsin B	1.46 for B-SiGMV, 3.67 for B-CuLCrV, −2.79 for Q-TYLCV
*Bta10767*	Cathepsin B	1.37 for B-CuLCrV, −1.35 for Q-TYLCV
*Bta12604*	Cathepsin B	−1.19 for B-SiGMV, −1.09 for Q-TYLCV
*Bta14750*	Cathepsin B	2.84 for B-CuLCrV −2.25 for Q-TYLCV
DEGs specific to MEAM1		
*Bta01559*	Aminopeptidase N, putative	−1.70 for TYLCV
*Bta03000*	Heat shock protein 70	−5.91 for CuLCrV
*Bta03004*	Suppressor of hairless protein	1.07 for TYLCV, 1.29 for SiGMV
*Bta03159*	Ras-like protein 2	1.17 for SiGMV
*Bta04167*	Ras-like GTP-binding protein RHO	1.22 for SiGMV
*Bta04210*	Protein kinase	1.60 for SiGMV
*Bta04481*	Calcium release-activated calcium channel protein 1	1.63 for SiGMV
*Bta04640*	Actin-related protein 2/3 complex subunit 2	1.38 for SiGMV
*Bta04715*	Ras-related C3 botulinum toxin substrate 1	1.69 for TYLCV, 1.49 for SiGMV
*Bta04792*	Aminopeptidase N-like protein	1.40 for TYLCV
*Bta04818*	Type I serine/threonine kinase receptor	1.47 for SiGMV
*Bta06076*	Heat shock protein	−1.39 for TYLCV
*Bta07992*	Protein kinase C	1.58 for SiGMV
*Bta08332*	Arrestin 1c	−1.89 for TYLCV
*Bta08697*	Cathepsin B	−1.17 for TYLCV
*Bta09313*	Cathepsin B	−2.34 for SiGMV
*Bta09601*	Unknown protein	−1.51 for TYLCV
*Bta10766*	Cathepsin B	1.02 for CuLCrV
*Bta11052*	Gelsolin	−1.19 for TYLCV
*Bta11333*	Peptidoglycan-recognition protein	−1.74 for TYLCV
*Bta11558*	Ras-related C3 botulinum toxin substrate 1	1.22 for TYLCV, 1.29 for SiGMV
*Bta12195*	Phosphoinositide phospholipase C	1.15 for SiGMV
*Bta12605*	Cathepsin B	1.31 for CuLCrV
*Bta12986*	Cathepsin B	−2.24 for TYLCV
*Bta13437*	Actin	−1.84 for TYLCV
*Bta14253*	Protein kinase C	1.54 for SiGMV
*Bta14532*	70 kDa heat shock protein	−2.20 for CuLCrV
*Bta14568*	Guanine nucleotide-binding protein G(S) subunit alpha	1.12 for TYLCV
*Bta14627*	Mothers against decapentaplegic homolog	1.15 for SiGMV
*Bta14721*	Cathepsin B	0.85 for CuLCrV
*Bta14751*	Cathepsin B, partial	1.32 for CuLCrV
*Bta15240*	F-box/WD repeat-containing protein 1A	1.19 for SiGMV
*Bta15478*	Ras-like GTP-binding protein RHO	1.45 for SiGMV
DEGs specific to MED		
*Bta00008*	70 kDa heat shock protein	−2.02 for SiGMV
*Bta01860*	Calreticulin	0.72 for TYLCV
*Bta02113*	Protein disulfide-isomerase	0.74 for TYLCV
*Bta02546*	Aminopeptidase-like protein	−0.72 for TYLCV
*Bta02612*	Protein kinase C	−0.82 for TYLCV
*Bta03883*	Cathepsin B	−1.42 for TYLCV
*Bta03885*	Cathepsin B	−1.36 for TYLCV
*Bta04776*	Cathepsin B	−1.26 for TYLCV
*Bta06903*	Cathepsin L	0.74 for TYLCV
*Bta08022*	CG13675, isoform D	−1.02 for SiGMV
*Bta08951*	Cathepsin B	−0.89 for TYLCV
*Bta09287*	Chaperone protein HtpG	0.73 for TYLCV
*Bta09613*	Cathepsin L, partial	2.92 for SiGMV
*Bta10363*	Cathepsin B	−0.93 for TYLCV
*Bta11419*	Cathepsin B	−1.34 for TYLCV
*Bta11420*	Cathepsin B	−1.45 for TYLCV
*Bta11544*	Heat shock 70 kDa protein 5	0.71 for TYLCV
*Bta12285*	Cathepsin B	−2.20 for TYLCV, −1.66 for SiGMV
*Bta12772*	Serine/threonine-protein phosphatase	−0.73 for TYLCV
*Bta14718*	Cathepsin B	−0.79 for TYLCV

Note. Genes with the same annotation name but different gene IDs are isoforms. B- and Q- represent the *B. tabaci* MEAM1 and MED.

**Table 5:** Differential expression of genes associated with cellular processes (apoptosis, lysosome, and phagosome) in viruliferous compared with non-viruliferous *Bemisia tabaci* Middle East-Asia Minor 1 (MEAM1) and Mediterranean (MED) adults.

Gene ID	Annotation	FC in Whitefly & Virus Treatment
DEGs specific to MEAM1		
*Bta01964*	Acid phosphatase-like protein	1.62 for TYLCV
*Bta02098*	Transport protein Sec61 subunit gamma	2.28 for SiGMV
*Bta02143*	Cathepsin F-like protease	−1.77 for TYLCV, −1.09 for SiGMV
*Bta03900*	Tubulin alpha-3 chain	−1.72 for TYLCV, −1.17 for SiGMV
*Bta04715*	Ras-related C3 botulinum toxin substrate 1	1.69 for TYLCV, 1.49 for SiGMV
*Bta04774*	Arylsulfatase	−1.44 for TYLCV
*Bta04870*	Cathepsin F	−1.89 for TYLCV
*Bta05911*	Cathepsin F-like protease	3.37 for CuLCrV
*Bta05912*	Cathepsin F-like protease	−2.06 for SiGMV
*Bta06085*	Protein kinase	−2.28 for TYLCV
*Bta06086*	Protein kinase C	−1.85 for TYLCV
*Bta06403*	Vesicular glutamate transporter 1	−1.44 for TYLCV
*Bta06690*	Cathepsin F	1.15 for CuLCrV
*Bta06693*	Cathepsin F	−1.05 for SiGMV
*Bta06964*	DNAation factor subunit beta	1.36 for SiGMV
*Bta07988*	Syntaxin-18	1.35 for SiGMV
*Bta07992*	Protein kinase C	1.58 for SiGMV
*Bta08697*	Cathepsin B	−1.17 for TYLCV
*Bta09313*	Cathepsin B	−2.34 for SiGMV
*Bta09676*	Cathepsin F	0.94 for CuLCrV
*Bta10683*	AP-1 complex subunit sigma-2	1.56 for TYLCV, 1.59 for SiGMV
*Bta10766*	Cathepsin B	1.02 for CuLCrV
*Bta11284*	Tetraspanin	−1.12 for TYLCV
*Bta11558*	Ras-related C3 botulinum toxin substrate 1	1.22 for TYLCV, 1.29 for SiGMV
*Bta11871*	Cathepsin F	−1.15 for TYLCV
*Bta12131*	Beta-hexosaminidase	−1.61 for TYLCV
*Bta12605*	Cathepsin B	1.31 for CuLCrV
*Bta12986*	Cathepsin B	−2.24 for TYLCV
*Bta13153*	Sensory neuron membrane protein 1	1.04 for SiGMV
*Bta13437*	Actin	−1.84 for TYLCV
*Bta14721*	Cathepsin B	0.85 for CuLCrV
*Bta14751*	Cathepsin B, partial	1.32 for CuLCrV
*Bta14774*	Beta-hexosaminidase	−2.13 for TYLCV
*Bta20004*	Cathepsin F	0.77 for CuLCrV
DEGs specific to MED		
*Bta01413*	Lipase	1.19 for TYLCV
*Bta01860*	Calreticulin	0.72 for TYLCV
*Bta03221*	Alpha-mannosidase	−0.72 for TYLCV
*Bta03883*	Cathepsin B	−1.42 for TYLCV
*Bta03885*	Cathepsin B	−1.36 for TYLCV
*Bta04776*	Cathepsin B	−1.26 for TYLCV
*Bta06903*	Cathepsin L	0.74 for TYLCV
*Bta07114*	Cathepsin F	−0.87 for TYLCV
*Bta07355*	N(4)-(Beta-N-acetylglucosaminyl)-L-asparaginase	1.06 for CuLCrV
*Bta08951*	Cathepsin B	−0.89 for TYLCV
*Bta09613*	Cathepsin L, partial	2.92 for SiGMV
*Bta10363*	Cathepsin B	−0.93 for TYLCV
*Bta10727*	Protein transport protein Sec61 subunit alpha isoform 2	0.92 for TYLCV
*Bta10829*	Acid phosphatase-1	−1.71 for TYLCV, 1.78 for SiGMV
*Bta11205*	Cathepsin F-like protease	−1.21 for CuLCrV
*Bta11419*	Cathepsin B	−1.34 for TYLCV
*Bta11420*	Cathepsin B	−1.45 for TYLCV
*Bta12285*	Cathepsin B	−2.20 for TYLCV, −1.66 for SiGMV
*Bta14718*	Cathepsin B	−0.79 for TYLCV

Note. Genes with the same annotation name but different gene IDs are isoforms. B- and Q- represent the *B. tabaci* MEAM1 and MED.

**Table 6:** Differential expression of genes associated with longevity in viruliferous compared with non-viruliferous *Bemisia tabaci* Middle East-Asia Minor 1 (MEAM1) and Mediterranean (MED) adults.

Gene ID	Annotation	FC in Whitefly & Virus Treatment
DEGs specific to MEAM1		
*Bta00981*	Cyclic AMP-responsive element-binding protein 3-like protein 2	1.60 for SiGMV
*Bta01299*	Forkhead box A	−2.21 for TYLCV
*Bta03000*	Heat shock protein 70	−5.91 for CuLCrV
*Bta03177*	Glutathione S-transferase	−1.31 for SiGMV
*Bta04098*	Acyl-CoA Z9 desaturase	−1.26 for SiGMV
*Bta06076*	Heat shock protein	−1.39 for TYLCV
*Bta06196*	Sestrin-like protein	1.19 for TYLCV, 1.30 for SiGMV
*Bta08630*	Fatty acyl-CoA reductase 1	1.30 for CuLCrV
*Bta08932*	Acyl-CoA desaturase 1	−1.63 for TYLCV
*Bta08933*	Acyl-CoA desaturase	3.55 for SiGMV
*Bta09151*	Protein kinase	−1.51 for TYLCV
*Bta13853*	Heat shock factor-binding protein, putative	2.42 for SiGMV
*Bta14222*	Fatty acyl-CoA reductase 1	−1.02 for TYLCV
*Bta14532*	70 kDa heat shock protein	−2.20 for CuLCrV
*Bta15447*	Glutathione s-transferase d1	−0.87 for SiGMV
DEGs specific to MED		
*Bta00008*	70 kDa heat shock protein	−2.02 for SiGMV
*Bta01763*	Acyl-CoA Delta (11) desaturase	0.95 for TYLCV
*Bta05672*	Acyl-CoA desaturase	−1.62 for TYLCV
*Bta11628*	Fatty acyl-CoA reductase 1	−1.58 for CuLCrV
*Bta13675*	Fatty acyl-CoA reductase 1	1.00 for TYLCV

Note. Genes with the same annotation name but different gene IDs are isoforms. B- and Q- represent the *B. tabaci* MEAM1 and MED.

## Data Availability

The raw read sequences were submitted to NCBI with the BioProject accession number PRJNA796977.

## Supplementary Materials

The following supporting information can be downloaded at: https://www.mdpi.com/article/10.3390/cells11132060/s1, Figure S1: TYLCV weighted gene co-expression network analysis. Figure S2: SiGMV weighted gene co-expression network analysis. Figure S3: CuLCrV weighted gene co-expression network analysis. Table S1: Endpoint PCR and qPCR primers used in this study. Table S2: Pearson’s correlation coefficients for multiple biological replicates. Table S3: RT-qPCR concordant validation for selected differentially expressed genes from RNA-Seq data. Table S4: KEGG annotations and pathway analysis for differential expressed genes in viruliferous versus non-viruliferous whiteflies.
