# Supplementary material for: Differential Transcriptional Responses in Two Old World Bemisia tabaci Cryptic Species Post Acquisition of Old and New World Begomoviruses
Source: Cells. 2022 Jun 29;11(13):2060. doi: 10.3390/cells11132060 (PMC9265393; doi:10.3390/cells11132060)
Supplement: Supplementary file 1 [file cells-11-02060-s001.zip › Figure S1-3.pptx]

## Slide 1
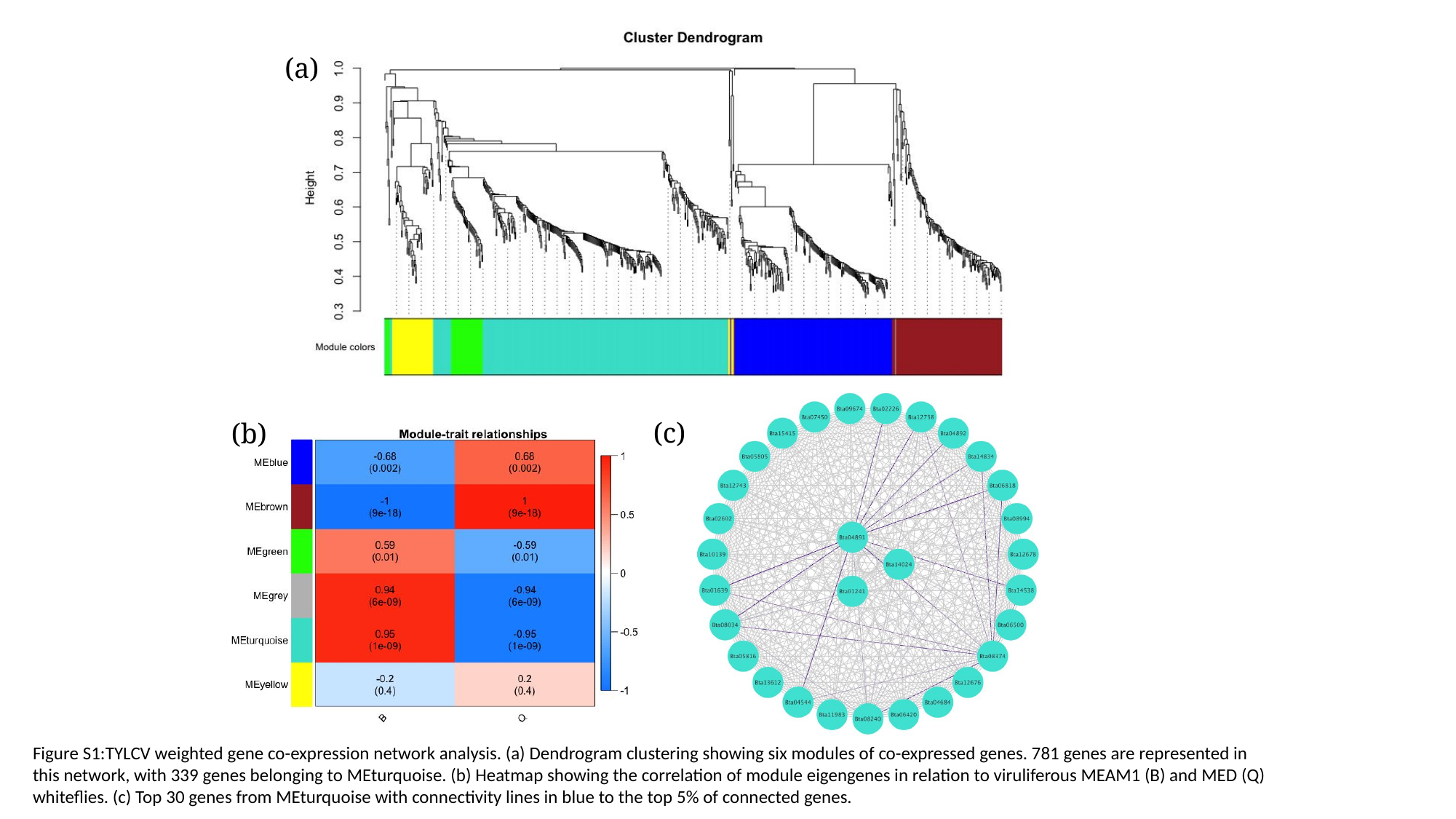

(a)
(c)
(b)
Figure S1:TYLCV weighted gene co-expression network analysis. (a) Dendrogram clustering showing six modules of co-expressed genes. 781 genes are represented in this network, with 339 genes belonging to MEturquoise. (b) Heatmap showing the correlation of module eigengenes in relation to viruliferous MEAM1 (B) and MED (Q) whiteflies. (c) Top 30 genes from MEturquoise with connectivity lines in blue to the top 5% of connected genes.

## Slide 2
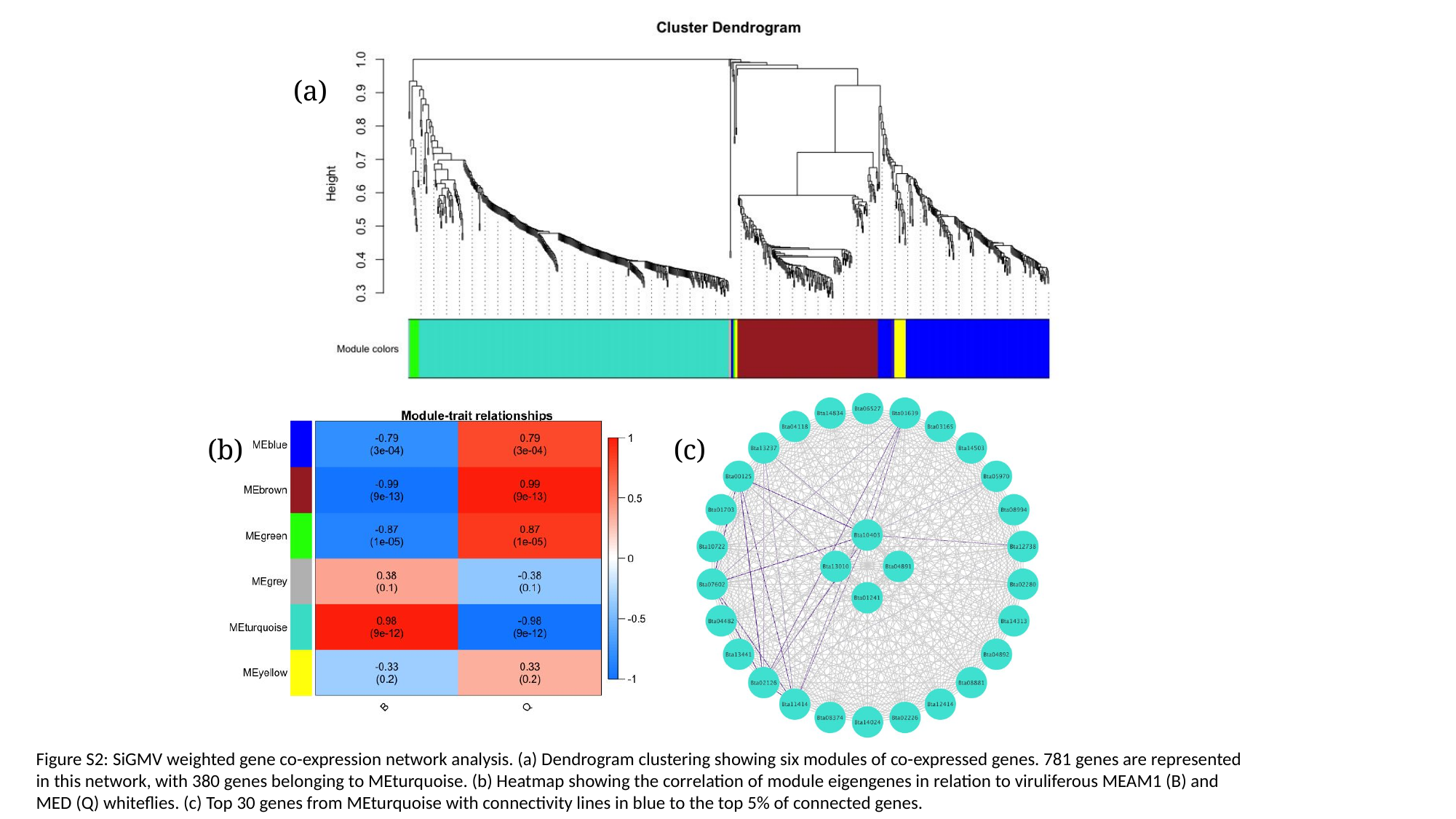

(a)
(b)
(c)
Figure S2: SiGMV weighted gene co-expression network analysis. (a) Dendrogram clustering showing six modules of co-expressed genes. 781 genes are represented in this network, with 380 genes belonging to MEturquoise. (b) Heatmap showing the correlation of module eigengenes in relation to viruliferous MEAM1 (B) and MED (Q) whiteflies. (c) Top 30 genes from MEturquoise with connectivity lines in blue to the top 5% of connected genes.

## Slide 3
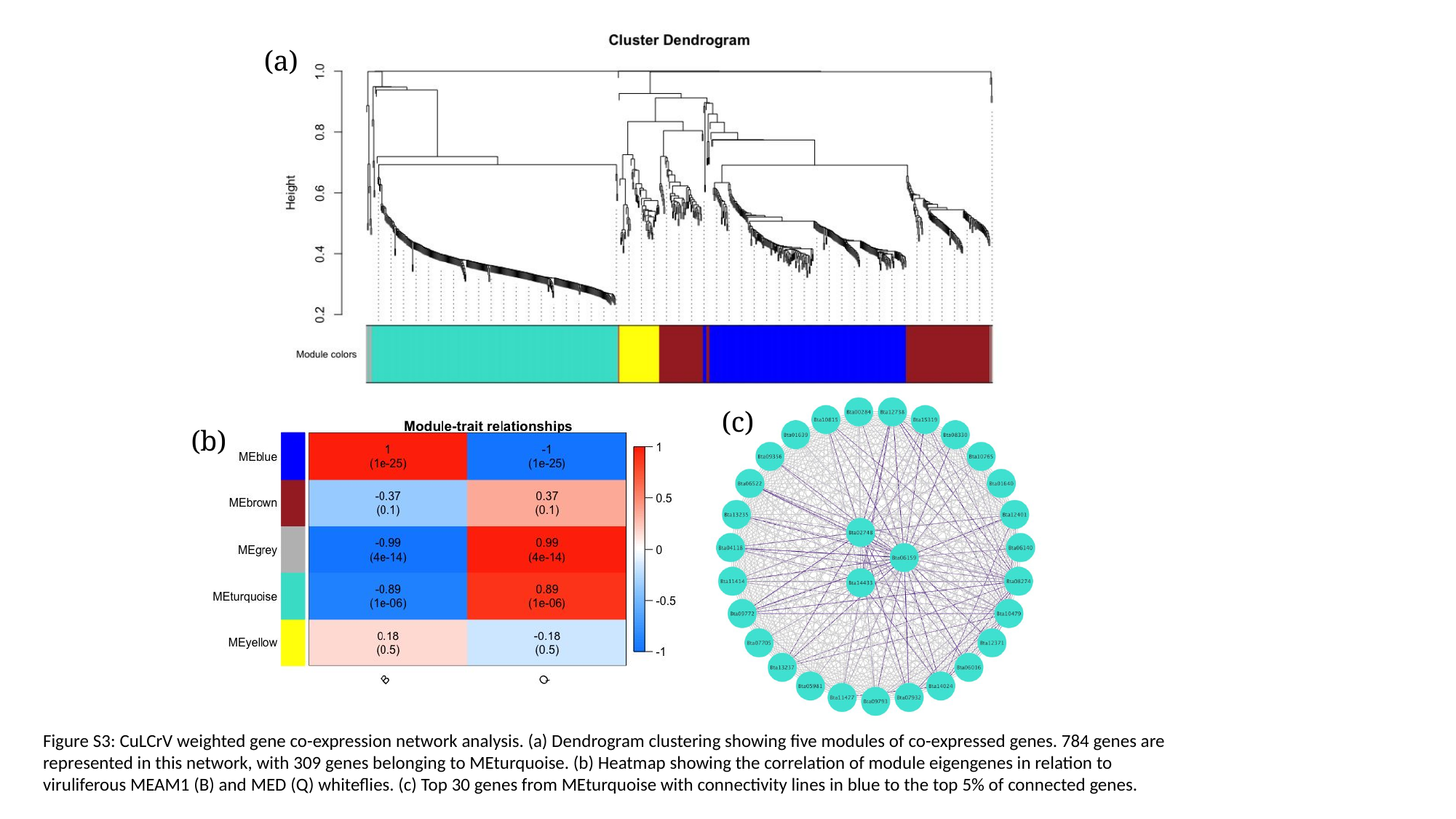

(a)
(c)
(b)
Figure S3: CuLCrV weighted gene co-expression network analysis. (a) Dendrogram clustering showing five modules of co-expressed genes. 784 genes are represented in this network, with 309 genes belonging to MEturquoise. (b) Heatmap showing the correlation of module eigengenes in relation to viruliferous MEAM1 (B) and MED (Q) whiteflies. (c) Top 30 genes from MEturquoise with connectivity lines in blue to the top 5% of connected genes.
